# Beef and coal are key drivers of Australia’s high nitrogen footprint

**DOI:** 10.1038/srep39644

**Published:** 2016-12-23

**Authors:** Xia Liang, Allison M. Leach, James N. Galloway, Baojing Gu, Shu Kee Lam, Deli Chen

**Affiliations:** 1 Crop and Soil Science Section, Faculty of Veterinary and Agricultural Sciences, The University of Melbourne, Victoria 3010, Australia; 2 Department of Natural Resources & Earth Systems Science and The Sustainability Institute, University of New Hampshire, 107 Nesmith Hall, 131 Main Street, Durham, NH, 03824, USA; 3 Department of Environmental Sciences, University of Virginia, Clark Hall, 291 McCormick Road, P.O. Box 400123, Charlottesville, VA 22904-4123, USA; 4 Department of Land Management, Zhejiang University, Hangzhou 310058, PR China

## Abstract

Anthropogenic release of reactive nitrogen (Nr; all species of N except N_2_) to the global nitrogen (N) cycle is substantial and it negatively affects human and ecosystem health. A novel metric, the N footprint, provides a consumer-based perspective for Nr use efficiency and connects lifestyle choices with Nr losses. Here we report the first full-scale assessment of the anthropogenic Nr loss by Australians. Despite its ‘clean and green’ image, Australia has the largest N footprint (47 kg N cap^−1^ yr^−1^) both in food and energy sectors among all countries that have used the N-Calculator model. About 69% of the Australia’s N footprint is attributed to food consumption and the associated food production, with the rest from energy consumption. Beef consumption and production is the major contributor of the high food N footprint, while the heavy dependence on coal for electricity explains the large energy N footprint. Our study demonstrates opportunities for managing Nr loss and lifestyle choices to reduce the N footprint.

More than half of the world’s population is nourished by food produced using synthetic nitrogen (N) fertilizers^1^,^2^. However, N escapes to the environment as reactive N (Nr; all species of N except N_2_) through different pathways during food and energy production and consumption^3^,^4^. With a large anthropogenic Nr creation rate and continued losses of Nr through human activities, increasing amounts of Nr are accumulating in the environment where they contribute to negative impacts. For example, the Nr-related damage in the European Union (EU) has been estimated at €70 billion to €320 billion per year^5^.

The N footprint is an index that assesses the total amount of Nr released to the environment as a result of an entity’s resource consumption^6^. It includes Nr losses from food production, food consumption, fossil fuel combustion for housing and transportation, and provision of goods and services. Using the N-Calculator model, the N footprint has been developed for many countries (i.e., United States, Netherlands, Germany, United Kingdom, Japan, Austria)^6^,^7^,^8^,^9^,^10^,^11^. This consumer-based tool can connect individual consumption choices with Nr losses and show how lifestyle choices affect these Nr losses. Using a different approach (a mass balance), N footprints have also been calculated in other countries such as China^12^. A Multi-Region Input-Output (MRIO) approach, which accounts for trade by tracking N losses through economic models, was used to calculate the N footprint for 188 countries^13^. However, compared to the N-Calculator, these approaches have less focus on consumers’ behavior.

While Australia and its products are often perceived as being ‘clean and green,’^14^ it has unique challenges for managing Nr issues. Although Australia is the planet’s sixth largest country, it has a small population (24 million) with a low population density of 3 people/km^2^ compared to the global average of 49 people/km^2^ (ref. 15). However, 85% of Australia’s population is found in the coastal areas, resulting in higher environmental risk to the coastal habitats (e.g., Great Barrier Reef)^16^. Furthermore, more than half of Australia’s land is used for farming production, 90% of which is used for grazing in the arid and semi-arid zones^17^. The widespread use of agricultural land in Australia has led to issues such as mining soil N in dryland rain-fed wheat systems and excessive N use in feedlot animal production systems^18^,^19^. Although numerous studies have linked agricultural Nr to environmental problems^20^,^21^,^22^, the N footprint has not been quantified for Australia. The objectives of our study were therefore to: (1) assess the Nr loss driven by food and energy consumption and associated production in Australia using the N-Calculator model; (2) benchmark Australia’s performance of Nr loss against other countries; and (3) explore the driving forces and mitigation strategies for the Australia’s N footprint.

## Results

### Per capita N footprint in Australia

**Figure 1:**
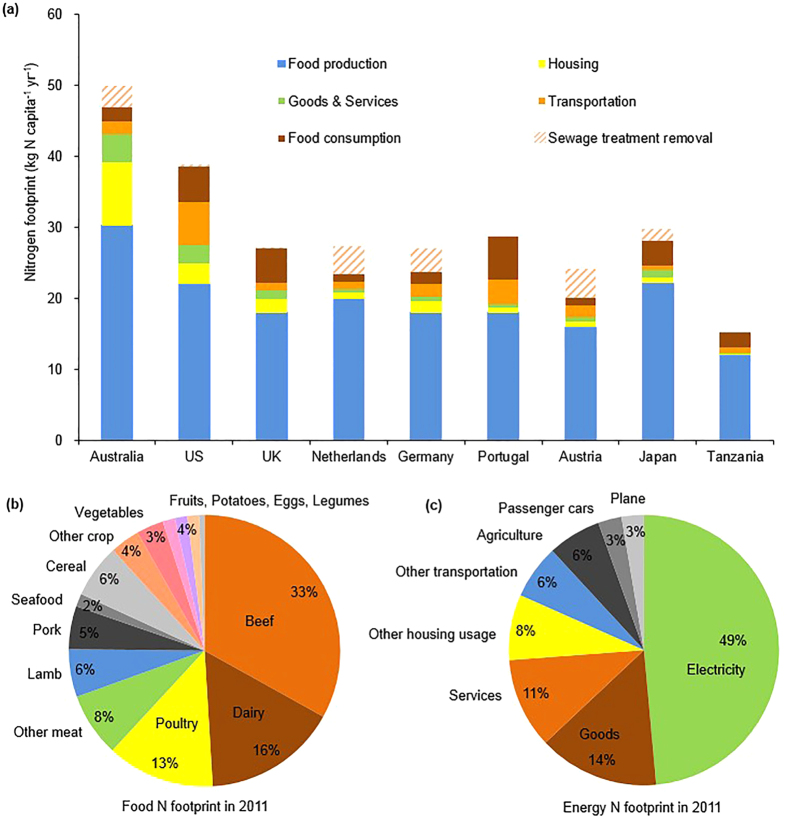
Components of Australia’s N footprint and comparisons with other countries. (**a**) Australia’s total N footprint in 2011 and comparisons with other countries. (**b**) The share of Nr emissions from the main food commodity groups (including consumption and production) in Australia. (**c**) The share of Nr emissions from the main energy sectors in Australia. “Sewage treatment removal” represents the part of food consumption Nr that is converted to N_2_ or recycled during sewage treatment.

The per capita N footprint in Australia was 47 kg N yr^−1^ in 2011. The food portion of the N footprint (32 kg N capita^−1^ yr^−1^) was the largest component, of which 2 kg N capita^−1^ yr^−1^ was from food consumption after sewage treatment and 30 kg N capita^−1^ yr^−1^ from the associated food production. However, before being corrected for the amount of Nr converted to N_2_ or recycled during sewage treatment, the actual food consumption N was 5 kg N capita^−1^ yr^−1^ (Fig. 1a, Supplementary Table 1). 82% of the food N footprint is from animal products, half of which is from beef (Fig. 1b). For crop products, cereals represent the largest proportion (35% of the crop N footprint), followed by vegetables, potatoes, fruits and legumes (Fig. 1b). The energy sectors contribute 15 kg N capita^−1^ yr^−1^, which is made up of the housing (9 kg N capita^−1^ yr^−1^), transportation (2 kg N capita^−1^ yr^−1^), and goods and services (4 kg N capita^−1^ yr^−1^) sectors (Fig. 1a, Supplementary Table 1). Almost half of the energy N footprint comes from electricity consumption (Fig. 1c).

### Australia’s virtual N factors (VNFs)

**Figure 2:**
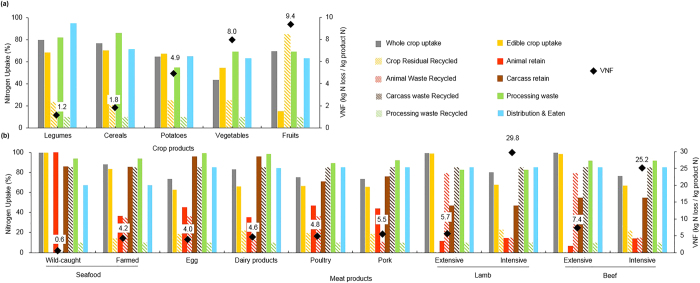
Nitrogen uptake (%) and virtual N factors (VNF) in Australia for the main food commodity groups by each step of the food production chain. (**a**) Vegetable products; (**b**) Animal products.

**Figure 3:**
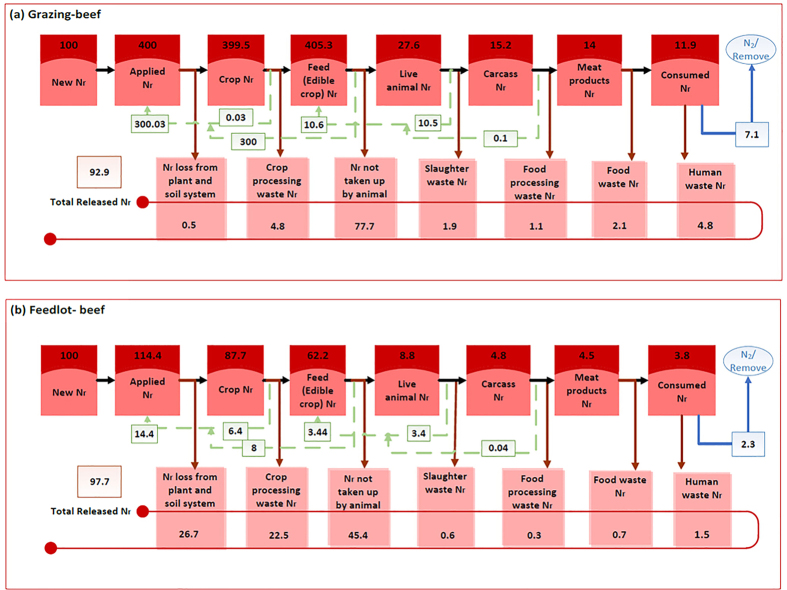
Reactive nitrogen (Nr) flows along the entire beef production and consumption chain in Australia. This explains the process used to calculate Nr flow in (**a**) a grazing system, starting with 100 units of new Nr; and (**b**) a feedlot system, starting with 100 units of new Nr. Notes: (1) The dark red boxes show the available Nr at each stage of the food production and consumption, with the numbers reflecting the magnitude of Nr; (2) The black arrows show the Nr that makes it to the next stage; (3) The brown arrows show the Nr that releases to the environment; (4) The light red boxes show the Nr loss at each stage of the food production and consumption, with the numbers reflecting the magnitude of Nr loss; (5) The transparent red boxes with number show the total Nr loss by all stages of food production and consumption; (6) The green dotted arrows show the Nr recycled; (7) The transparent green boxes with numbers show the amount of recycled Nr which is subtracted from the Nr wasted to find the actual Nr lost to the environment; (8) The blue arrows show the consumed Nr that is converted to N_2_ or recycled during sewage treatment; (9) The transparent blue boxes with number show the amount of consumed Nr that is converted to N_2_ or recycled during sewage treatment; and (10) The diagrams show the summation of multiple iterations of the calculations; the iterations determine how recycled Nr is distributed throughout the system.

There is a large variability in Australia’s VNFs (a metric that describes the total Nr loss to the environment during food production per unit of N in the consumed food product^6^), which range from 1.2 (legumes) to 29.8 (feedlot lamb) kg N released to the environment per kg N consumed (Table 1, Fig. 2, Supplementary Table 4). Generally, animal products’ VNFs are larger than those of plant products. For plant products, the VNF is as low as 1.2 (legumes) and 1.8 (cereals), and as high as 8.0 (vegetables) and 9.4 (fruits). The VNF of animal products ranges from 0.6 for wild-caught seafood to 25.2 and 29.8 for feedlot beef and lamb respectively; other meat products have a VNF of around 5 (Table 1, Fig. 2, Supplementary Table 4). Production methods affect the VNFs. Examples include wild-caught seafood (0.6) vs. farmed seafood (4.2), lamb from the grazing system (5.7) vs. feedlot system (29.8), and beef from grazing system (7.4) vs. feedlot system (25.2) (Fig. 2b, Supplementary Table 4). Australia’s feedlot production systems have a higher VNF than grazing production systems (Fig. 3). Although most of Australia’s sheep and cattle by count are grazing outdoors, the feedlot systems contribute a large percentage of meat production. The Australian beef and lamb VNFs weighted by production are still higher than those of other countries.

### Australia vs. other countries

At a global scale, Australia has the largest N footprint both in the food and energy sectors among the nine countries where the N footprint has been calculated using the N-Calculator model (Fig. 1a, Supplementary Table 1). Australia’s food N footprint is the largest because it has the highest rate of beef consumption and the highest beef VNF (Fig. 1a, Table 1, Supplementary Table 1). The food N footprint is the largest component of the total N footprint among all of the countries (69–92% of the total N footprint), and the amount of Nr loss associated with food production is the highest in Australia (30 kg N capita^−1^ yr^−1^). The amount of N consumed as food in Australia (5 kg N capita^−1^ yr^−1^) is similar to that in other developed countries (5–6 kg N capita^−1^ yr^−1^) but higher than that in less-developed countries (Tanzania; 2 kg N capita^−1^ yr^−1^). However, Australia and most of the developed countries use advanced wastewater treatment to recycle or convert Nr to N_2_, which substantially diminishes the discharge of Nr from food consumption to the environment (e.g. the Nr removal factor is 60% in Australia, 79% in Austria, 78% in the Netherlands, and 67% in Germany).

For the energy sectors, Australia has the highest total energy N footprint (15 kg N capita^−1^ yr^−1^) among all countries, which is several times higher than the Netherlands, Austria, Japan, and Germany (2–4 kg N capita^−1^ yr^−1^) and even tenfold higher than Tanzania (1 kg N capita^−1^ yr^−1^) (Fig. 1a, Supplementary Table 1). Australia’s energy N footprint is the largest due to a heavy dependence on coal, which drives up its electricity N footprint.

## Discussion

The VNFs, which represent the Nr losses along the food production chain, vary substantially with food types^6^. In most countries, production of legumes and seafood is the most efficient while the production of beef is the least efficient in terms of Nr use^6^,^7^,^8^,^9^,^23^ (Table 1, Supplementary Table 4). Although vegetables and fruits have large VNFs because of low N use efficiency in these production systems^24^,^25^, the Nr loss per serving of vegetables or fruits is low due to the very low N content compared to other food types^9^. Beef production is the least efficient way of using N and supplying dietary protein in most countries that have completed their calculation of VNFs^6^,^7^,^8^,^9^,^23^, mainly due to the large feed requirements and their high basal metabolic rate^26^. An analysis of the environmental impacts of the various livestock categories in the United States had a consistent finding^6^,^8^,^27^. Eshel *et al*.^27^ demonstrated that in addition to higher Nr losses, per consumed calorie of beef also required more land and irrigation water and released more GHG than the other livestock products.

The VNFs also vary significantly across nations due to different food production methods. Australia has lower VNFs than Japan but higher VNFs than other countries that have completed this calculation (Table 1). The key variables that affect these results are the fertilizer N use efficiency for crops and the feed N conversion ratio for animals. For instance, the portion of N uptake from fertilizer by cereals is 80% in the US and 43% in Japan, and the portion of N retained in feedlot cattle is 20% in the US and 14% in Japan^6^,^8^. The Australian value for N uptake from fertilizer by cereals (77%) is comparable to the US but much higher than Japan, whereas the value for N retained in feedlot cattle (14%) is the same as Japan, but much lower than the US. These values drive the VNFs and suggest that less Nr is released from cereal production in Australia than in Japan, but more Nr from feedlot beef production in Australia than in the US. However, VNF calculation looks just at the Nr loss during production and does not connect to environmental impacts. Because of the extensive grazing in Australia, the high Nr losses from beef could be diluted by the extensive space and arid climate.

According to the Australia’s Dietary Guidelines (ADGs), the recommended consumption of high protein food (lean meat, fish, and eggs) is 200–300 g capita^−1^ day^−1^ (for the age group 19–50 years), but the actual consumption level is double^28^,^29^. Meanwhile, the actual consumption of cereals (240 g capita^−1^ day^−1^) is much lower than the recommended amount of 600 g capita^−1^ day^−1^ (ref. 29). The required annual N intake as protein is 2.5–3.5 kg N capita^−1^ based on the WHO^30^ and USDA^31^ dietary recommendation, but Australians consume 5.0 kg N capita^−1^ yr^−1^ (Fig. 1a, Supplementary Table 1). 72% of N consumption in Australia is from animal food (Table 1), and beef is the main contributor. Beef consumption by Australia’s people is more than double and four times larger than the consumption by the British and the Japanese, respectively (Table 1). The high consumption level and high VNF of beef resulted in Australia having the largest beef N footprint (11.1 kg N capita^−1^), which is much greater than that in other countries (Table 1). As studies have shown how food choices and diet changes can reduce the Nr emission in the European Union^32^,^33^, choosing a diet better aligned with the Australia dietary guidelines would both reduce Australia’s N footprint and improve consumers’ health.

Australia has a large land area with low rainfall and fertility, both of which promote the long tradition of grazing cattle. About 97–98% of 28 million cattle in Australia are located in extensive native grasslands^34^, which receive little or no fertilizer N^35^. Although the N feed conversion ratio by feedlot cattle (14%)^36^ is higher than cattle in extensive grazing farms (7–10%)^37^,^38^, we found that 79% of N excreted by grazing cattle will return to the grassland whereas only 15% of N excreted from feedlot cattle will be collected and reused (Fig. 2b, Fig. 3, Supplementary Fig. 1, Supplementary Table 4). In particular, ammonia (NH_3_) emissions from the grazing system are estimated at 0.2 kg NH_3_-N/kg manure N^39^ but 0.81 kg NH_3_-N/kg manure N in the feedlots^21^,^36^. That means that when producing the same amount of beef, more Nr is released to the environment from feedlot systems than from grazing farms. Likewise, the comparisons between grazing versus feedlot lamb and wild-caught versus farmed seafood also indicate that the extensive food production systems in Australia release less Nr than the intensive ones (Fig. 2b, Supplementary Fig. 1, Supplementary Table 4). Around two thirds of total Australia’s agricultural products are exported worldwide with an international reputation for clean and green production^40^. In particular, the relatively mild climate enables livestock to graze year-round^41^, which supports protein-rich diets in Australia and helps to produce large exportable food surpluses (e.g., Australia is the second largest exporter of beef in the world)^42^.

Australia also has abundant energy resources. Due to the plentiful resources and low-cost production^43^, 69% of electricity was generated from coal during 2012–2013, which was higher than any other developed country (e.g., 43% in the US and 29% in the UK)^44^. We demonstrated that Australia emitted about 7.5 kg N capita^−1^ from electricity generation in 2011, compared to 3.7 and 1.8 kg N capita^−1^ for the US and the UK, respectively. The high N emissions associated with electricity generation concomitantly contributed to a larger N footprint for goods and services in Australia (Fig. 1a, Supplementary Table 1). The high transportation N footprint is caused by the low housing density and long distances of daily travel in Australia, as well as the less stringent regulations on energy efficiency, vehicle emissions and carbon pricing than other developed nations such as the US and the UK^45^. However, Australia has a rich diversity of renewable energy resources (wind, solar, geothermal, hydro, wave, tidal, bioenergy), the wide adoption of these renewable resources would decrease the energy component of the N footprint.

The unexpectedly large Australian N footprint has not been uncovered until now. Our results show that lifestyle choices (especially beef consumption and coal for electricity) have major impacts on Nr losses to the environment. Consumers could reduce their N footprint by choosing a diet with less meat and using more renewable energy. The on-farm N footprint can be reduced by improving farm N use efficiency, such as by the 10 “Key Actions” listed in “Our Nutrient World”^46^. These strategies are critical for meeting the increasing demand for food and energy in an environmentally sustainable way.

## Materials and Methods

### Study area

This consumer-based study accounts for the all domestic consumption and the associated production of food and energy in Australia. Given this consumer focus, this study does not account for the N footprint of exports. We also assumed that all food and energy were produced using Australia’s production methods and therefore did not account for the effects of imports. The N footprint was divided into food, housing, transportation and goods & services, and each sector was further divided into subsectors to cover the major human activities related to Nr loss.

### N footprint calculation

We followed the methodology of the N-Calculator model proposed by Leach *et al*.^6^, which was also adopted by the Netherlands^6^, Germany^7^, UK^7^, Austria^9^, Japan^8^, and Tanzania^23^.

For the food component of the N footprint, we estimated the Nr losses to the environment along the food production and consumption chain, starting with N fertilizer applied to cropland and ending with sewage treatment. The food N footprint has two parts: the food consumption N footprint and the associated food production N footprint. Food consumption was calculated by subtracting food waste from the Australia’s food supply^28^. The food consumption N was assumed to be completely excreted since adults generally do not accumulate N in their body^47^. Nonetheless, the widespread use of advanced sewage treatment in Australia removes 60% of this Nr from the sewage stream by either converting it into a non-reactive form (N_2_) or recycling and reusing it in the form of sludge^48^,^49^. The N removal from the sewage stream is considered a reduction to the food consumption N footprint.

The associated food production N footprint accounts for all N lost during the food production process. The food production N footprint is calculated using virtual N Factors (VNFs), which are defined as the total Nr loss to the environment during production per unit of N in the final consumed food products. Examples of these N losses include the fertilizer Nr not incorporated into the plant, feed Nr not retained in the animal products, crop residues Nr not recycled, and processing and food waste Nr^6^. Food production N is calculated by multiplying the VNF by the food N consumed; the VNFs covers all steps from initial fertilizer application to final food consumption. We calculated Australia’s VNFs for 12 major food categories: cereals (weighted-average of rice, wheat, barley, sorghum), legumes, potatoes, vegetables, fruits, seafood (weighted-average of wild-caught and farmed), poultry, egg, dairy products (weighted-average of milk, cheese, yoghurt and dry milk), pork, lamb and beef (weighted-average of grazing and feedlot systems) (see Supplementary Table 4, Supplementary Fig. 1 for Nr flow along the food production and consumption chain).

Nr losses associated with energy use during food production (e.g., on-farm energy use, food processing, packaging, transportation) were also included as part of the food N footprint. We used an environmentally extended input–output analysis (EEIO) to allocate Australia’s total NO_x_ and NH_3_ emissions associated with food production to various food categories^6^,^50^.

The energy component of the N footprint consists of four main aspects of daily life associated with energy consumption: housing (e.g., electricity, natural gas use), transportation (e.g., plane, train, car), goods (e.g., clothing, furniture, tools) and services (e.g., education, insurance and financial services). A bottom-up approach was adopted to calculate the N footprint for major areas of N consumption (electricity, heating, personal travel). For these sectors, activity data (e.g. hours flown by aircraft) was used with an emission factor (e.g. amount of Nr emitted per hour flying) (see Supplementary Table 3 for data and references). A top-down approach (EEIO analysis for emissions of N from NO_x_ and NH_3_ within Australia) was used to calculate the N footprint of the remaining sectors related to consumers^6^,^50^. After removing any double-counting of fuel sources, the sum of the bottom-up and top-down footprints is the total energy N footprint.

### Data sources

Key sources of data for the N footprint included: International statistical data sources; Australia’s governmental statistical data sources; industry data sources; data from a number of published articles for some coefficients; consultation with industry, researchers, and other experts in the field (Supplementary Tables 2, 3 and 4).

## Additional Information

**How to cite this article**: Liang, X. *et al*. Beef and coal are key drivers of Australia’s high nitrogen footprint. *Sci. Rep.* **6**, 39644; doi: 10.1038/srep39644 (2016).

**Publisher's note:** Springer Nature remains neutral with regard to jurisdictional claims in published maps and institutional affiliations.

## Supplementary Material

Supplementary Information

## Figures and Tables

**Table 1:** Comparisons of the virtual N factors (VNFs; units: kg N loss (kg consumed N)^−1^), N consumption (kg N capita^−1^ yr^−1^) and N footprints (kg N capita^−1^ yr^−1^) for major food categories in Australia and other countries.

Products	Cereals	Legumes	Potatoes	Vegetables	Fruits	Seafood	Egg	Poultry	Dairy	Pork	Lamb	Beef
**Virtual N factors**												
Australia	1.8	1.2	4.9	8.0	9.4	1.9	4.0	4.8	5.0	5.5	9.3	13.4
US^6^	1.4	0.5	1.5	9.6	9.6	4.1	3.2	3.2	4.3	4.4	5.2	7.9
UK^7^	1.3	0.5	1.1	8.2	8.2	2.9	3.2	3.2	3.9	4.4	5.2	7.9
Austria^9^	1.2	0.4	2.0	4.3	4.3	—	2.5	2.5	3.7	3.6	3.8	5.4
Japan^8^,*	3.3	2.8	6.1	4.6	4.6	1.7	10.7	10.7	3.9	12.9	5.6	27.3
Tanzania^23^	6.3	0.3	1.8	4.1	4.1	0.2	0.5	0.8	8.3	3.3	3.3	7.0
**Nitrogen consumption**^28^												
Australia	0.86	0.13	0.08	0.11	0.04	0.21	0.10	0.78	0.94	0.27	0.19	0.77
US	0.98	0.11	0.10	0.11	0.05	0.19	0.20	0.92	1.10	0.37	0.01	0.63
UK	1.21	0.08	0.18	0.14	0.06	0.26	0.16	0.62	1.04	0.37	0.08	0.33
Austria	1.15	0.03	0.10	0.15	0.08	0.18	0.21	0.40	1.12	0.93	0.02	0.35
Japan	1.01	0.06	0.04	0.17	0.03	0.89	0.30	0.34	0.39	0.28	0.01	0.18
Tanzania	1.25	0.65	0.21	0.07	0.08	0.10	0.01	0.02	0.18	0.01	0.03	0.12
**Nitrogen footprints**												
Australia	2.41	0.28	0.47	1.03	0.42	0.61	0.52	4.54	5.66	1.78	1.93	11.07
US	2.36	0.17	0.25	1.22	0.51	0.94	0.86	3.88	5.81	2.02	0.05	5.60
UK	2.77	0.12	0.39	1.29	0.59	1.00	0.69	2.62	5.11	1.99	0.49	2.98
Austria	2.53	0.04	0.31	0.79	0.41	—	0.75	1.40	5.26	4.29	0.09	2.24
Japan	4.35	0.21	0.27	0.95	0.15	2.40	3.49	4.02	1.92	3.93	0.02	5.00
Tanzania	9.14	0.85	0.59	0.35	0.40	0.12	0.02	0.04	1.66	0.02	0.11	0.98

^*^Japan without trade.
