# Supplementary material for: Beef and coal are key drivers of Australia’s high nitrogen footprint
Source: Sci Rep. 2016 Dec 23;6:39644. doi: 10.1038/srep39644 (PMC5180353; doi:10.1038/srep39644)
Supplement: Supplementary Information [file srep39644-s1.docx]

**Supplementary Information**

**Beef and coal are key drivers of Australia's high nitrogen footprint**

Xia Liang^a^, Allison M. Leach^b^, James N. Galloway^c^, Baojing Gu^a,d^, Shu Kee Lam^a^, Deli Chen^a*^

^a^ Crop and Soil Science Section, Faculty of Veterinary and Agricultural Sciences, The University of Melbourne, Victoria 3010, Australia.

^b^ Department of Natural Resources & Earth Systems Science and The Sustainability Institute, University of New Hampshire, 107 Nesmith Hall, 131 Main Street, Durham, NH, 03824, USA.

^c^ Department of Environmental Sciences, University of Virginia, Clark Hall, 291 McCormick Road, P.O. Box 400123, Charlottesville, VA 22904-4123, USA.

^d^ Department of Land Management, Zhejiang University, Hangzhou 310058, PR China

***Corresponding Author:**

Deli Chen

Professor and Head of Crop and Soil Science, Faculty of Veterinary and Agricultural Sciences, The University of Melbourne, Victoria 3010, Australia.

E-mail: delichen@unimelb.edu.au | Tel.: +61 3 8344 8148.

**Supplementary Method**

***Australian virtual N factors (VNFs)***

We initiate a whole set of Australian VNFs for 12 major food categories: cereals (weighted-average of rice, wheat, barley and sorghum), legumes, potatoes, vegetables, fruits, seafood (weighted-average of wild-caught and farmed), poultry, egg, dairy products (weighted-average of milk, cheese, yoghurt and dry milk), pork, lamb and beef (weighted-average of grazing and feedlot system), and assigned other minion food categories to the most similar major one above.

To get each VNF, 6 ratio coefficients for crop products and 10 ratio coefficients for meat products are required, respectively listing as: (1) % of available N uptake by whole plant (2) % of whole plant N retained in edible part (3) % of residual N recycled (4) % of edible crop N produced as food products (5) % of processing waste N recycled (6) % of crop products N consumed finally; (1) % of available N uptake by whole plant (2) % of whole plant N retained in animal feed (3) % of residual N recycled (4) % of feed N assimilated by live animals (5) % of feed waste and excreted N recycled (6) % of live animals N stayed in carcasses (7) % of slaughter waste N recycled (8) % of carcasses N produced as meat products (9) % of processing waste N recycled (10) % of meat products N consumed finally. We also combined the ^15^N tracer studies, N mass balance approach and life cycle analysis (LCA) to make the best assessment with Australian limited research and data source. Nr flows along the entire production and consumption chain of 11 major food categories in Australia are shown in Table S 4 and Fig S1.

**Limitations and uncertainties**

As with any modelling calculation, there are uncertainties associated with the data sets and results of a given country’s N footprint and when comparing across countries. To reduce uncertainty in comparisons, an established methodology from Leach et al. (2012) is used for all country N footprint calculations. This methodology describes the appropriate data sets and equations used to calculate a country’s N footprint. For a given country’s calculation, the data sets that are used range in their level of certainty; it’s clear that the uncertainty in some of the data source is larger than for others among nations. For example, data on food consumption is reported by countries in an international database through FAOSTAT^1^. This provides a consistent data set available for all countries with consistent assumptions. Fertilizer application and nitrogen use efficiency estimates for the virtual N factor calculations, however, rely on national statistics, which can lead to some inconsistencies in the assumptions. Data sets are selected so that they most closely match the assumptions and methodology for N footprint calculations. Despite the uncertainty associated with some of the data sets, our analysis is based on the best data currently available and provides a reliable footprint result and comparisons.

**Supplementary Table 1: Australian N footprint and the comparison with other countries.**

|  | Australia | US[^1^](#_ENREF_1) | UK[^2^](#_ENREF_2) | Netherlands[^1^](#_ENREF_1) | Germany[^2^](#_ENREF_2) | Portugal[^3^](#_ENREF_3) | Austria[^4^](#_ENREF_4) | Japan[^5^](#_ENREF_5) | Tanzania[^6^](#_ENREF_6) |
| --- | --- | --- | --- | --- | --- | --- | --- | --- | --- |
| Food production | 30.3 | 22.0 | 18 | 20.0 | 18.0 | 18.0 | 16.0 | 22.2 | 12.0 |
| Housing | 8.8 | 3.0 | 2.0 | 0.8 | 1.6 | 0.7 | 0.8 | 0.8 | 0.2 |
| Goods & Services | 3.9 | 2.5 | 1.1 | 0.5 | 0.7 | 0.5 | 0.6 | 1.0 | 0.2 |
| Transportation | 1.9 | 6.0 | 1.1 | 1.1 | 1.8 | 3.5 | 1.6 | 0.7 | 0.8 |
| Food consumption | 2.0 | 5.0 | 4.9 | 1.1 | 1.6 | 6.0 | 1.1 | 3.4 | 2.0 |
| Food consumption, denitrified /recycled | 3.0 | 0.3 | 0.1 | 3.9 | 3.3 | 0.0 | 4.1 | 1.7 | 0.0 |
| Total | 46.9 | 38.5 | 27.1 | 23.5 | 23.7 | 28.7 | 20.1 | 28.1 | 15.2 |

**Supplementary Table 2: Key data sources for calculating the nitrogen footprint of Australia.**

| **Key data sources** | **Data collected** |
| --- | --- |
| **International statistical data** |  |
| UN Food and Agricultural Organization (FAO) | Food supply and protein supply data by all food types[^7^](#_ENREF_7) |
| International Fertilizer Industry Association | Nitrogen fertilizer use data[^8^](#_ENREF_8) |
| Global Food Losses and Food Waste | Global food waste data by each food category[^9^](#_ENREF_9) |
| International Plant Nutrition Institute (IPNI) | Estimates of Nutrient Uptake and Removal[^10^](#_ENREF_10) |
| **Australia’s governmental statistical data** |  |
| Australia’s Bureau of Agricultural and Resource Economics | Australia agricultural independent research data[^11^](#_ENREF_11) |
| Australia’s Bureau of Statistics | Australia national and regional statistics data based on surveys and census[^12-14^](#_ENREF_12) |
| National Health and Medical Research Council | Australia’s Dietary Guidelines and food portion size data[^15^](#_ENREF_15) |
| Bureau of Resources and Energy Economics | Australia’s resource and energy use data[^16^](#_ENREF_16) |
| Council of Australia’s Governments | Governmental criteria data[^17^](#_ENREF_17) |
| Department of Infrastructure and Regional Development | National and regional energy statistics data[^18^](#_ENREF_18) |
| Department of Industry | Industrial criteria data[^19^](#_ENREF_19) |
| Australia’s Automobile Association | Australia’s automobile statistics data[^20^](#_ENREF_20) |
| Bureau of Infrastructure, Transport and Regional Economics | Australia’s transportation statistics data |
| Department of Infrastructure and Transport | Australia’s transportation statistics data[^21^](#_ENREF_21) |
| Climate Change Authority | Australia’s automobile emmission criteria data[^22^](#_ENREF_22) |
| Department of Climate Change and Energy Efficiency | Australia’s automobile emmission factors data[^23^](#_ENREF_23) |
| Department of the Environment | National Nr emission estimates that under the United Nations Framework Convention on Climate Change (UNFCCC) and the Kyoto Protocol (KP)[^24^](#_ENREF_24) |
| **Industry data** |  |
| Water Services Association of Australia | National water and sewerage services data[^25^](#_ENREF_25) |
| Meat and Livestock Australia | Australia’s cattle, sheep husbandry and meat processing data[^26^](#_ENREF_26) |
| Australia’s Pork Limited | Australia’s pig raise and pork production data[^27^](#_ENREF_27) |
| Dairy Australia | Australia’s dairy industry data[^28^](#_ENREF_28) |
| National Seafood Industry Alliance | Australia’s wild-caught fishing and aquaculture industries data[^29^](#_ENREF_29) |
| Australia’s Egg Corporation Limited | Australia’s egg industry data[^30^](#_ENREF_30) |
| Australia’s Chicken Meat Federation | Australia’s poultry industry data[^31^](#_ENREF_31) |
| **Published articles** | Nitrogen fertilizer recovery data; harvest index data and waste recycle data[^10^](#_ENREF_10)^,^[^14^](#_ENREF_14)^,^[^32^](#_ENREF_32)^,^[^33^](#_ENREF_33) |
| **Consultation with industry and the personal knowledge of the researchers for the unpublished data** | Domestic aviation and rail emission factors, N fertilizer usage and recovery, N retention during animal husbandry |

**Supplementary Table 3: Data and references used for the calculation of the average per capita energy N footprint: housing, transportation, goods & services.**

| **Housing** | | **Average consumption^a^** | **Energy content factor** | **Emission factors^b^** | |
| --- | --- | --- | --- | --- | --- |
| Electricity | | 605.39 kWh/household/month[^16^](#_ENREF_16) | - | 0.002749kg N/kWh (ref. [^34-36^](#_ENREF_34)) | |
| Natural gas | | 36.69 m^3^/ household/month[^16^](#_ENREF_16) | 39.3×10^-3^ GJ/m^3^ (ref. [^37^](#_ENREF_37)) | 0.000457 kg N /m^3^ (ref. [^24^](#_ENREF_24)) | |
| LPG | | 5.91 l/ household/month[^16^](#_ENREF_16) | 25.7 GJ/kL (ref. [^37^](#_ENREF_37)) | 0.000517 kg N /l (ref. [^24^](#_ENREF_24)) | |
| Wood& Woodwaste | | 31.75 kg/ household/month[^16^](#_ENREF_16) | 16.2 GJ/t (ref. [^37^](#_ENREF_37)) | 0.000411 kg N /kg (ref. [^24^](#_ENREF_24)) | |
| Solar energy | | 109.62 MJ/ household/month[^16^](#_ENREF_16) | - | - | |
| **Number of people per household** | | 2.63 (ref. [^38^](#_ENREF_38)) | **Average household income** | 918 $/ week (ref. [^39^](#_ENREF_39)) | |
| **Transportation** | | **Average consumption^a^** | | | |
| Plane | | 2889.74 passenger-km/person/year[^18^](#_ENREF_18)^,^[^21^](#_ENREF_21)^,^[^40-42^](#_ENREF_40) | | | |
| Rail | | 12.89 passenger-km/person/week[^18^](#_ENREF_18)^,^[^21^](#_ENREF_21)^,^[^40-42^](#_ENREF_40) | | | |
| Passenger cars | | 144.64 km/person/week[^43^](#_ENREF_43)^,^[^44^](#_ENREF_44) | 0.0000585 kg N/km[^24^](#_ENREF_24)^,^[^40-42^](#_ENREF_40) | | |
| Light commercial vehicles | | 37.11 km/person/week[^21^](#_ENREF_21)^,^ [^22^](#_ENREF_22) | 0.0000585 kg N/km[^12^](#_ENREF_12)^,^ [^15^](#_ENREF_15)^,^ [^16^](#_ENREF_16)^,^ [^19^](#_ENREF_19) | | |
| Motor cycles | | 2.34 km/person/week[^21^](#_ENREF_21)^,^ [^22^](#_ENREF_22) | 0.0000652 kg N/km[^24^](#_ENREF_24)^,^[^40-42^](#_ENREF_40) | | |
| Buses | | 1.95 km/person/week[^43^](#_ENREF_43)^,^[^44^](#_ENREF_44) | 0.0003659 kg N/km[^24^](#_ENREF_24)^,^[^40-42^](#_ENREF_40) | | |
| Rigid trucks | | 7.93 km/person/week[^21^](#_ENREF_21)^,^ [^22^](#_ENREF_22) | 0.0003659 kg N/km[^24^](#_ENREF_24)^,^[^40-42^](#_ENREF_40) | | |
| **Goods & Services** |  | | | | |
| Goods | | 2.26 kg N /person/year[^45^](#_ENREF_45) | | |  |
| Services | | 1.69 kg N /person/year[^23^](#_ENREF_23)^,^ [^24^](#_ENREF_24) | | |  |

Notes: ^a^: ABS Energy Accounts data for total net energy use for households includes energy used by households for transport. BREE Energy Balances data assigns all energy used in transport, including that used by the Residential sector, to the Transport sector. Therefore, the total net energy usage for households reported by BREE is substantially lower than ABS estimates. (ABS Energy Accounts data for 2011: Electricity-572.7 kWh/month/household, Natural gas-37.05m3/month/household, Petrol-137.13 l/month/household, LPG-14.21 l/month/household, Diesel-22.46 l/month/household, Renewable energy (biomass)-22.67 l/month/household).

^b^: These emission factors describe the NO_X_ emitted, converted to units of N assuming all NO_X_ is NO_2_.

**Supplementary Table 4: Nitrogen uptakes (%) and VNFs for the main food commodity groups by each step and key references.**

|  | | Legumes | | Cereals | | | Potatoes | | | Vegetables | | Fruits | | |  |  |
| --- | --- | --- | --- | --- | --- | --- | --- | --- | --- | --- | --- | --- | --- | --- | --- | --- |
| Whole crop uptake | | 80% [^46-49^](#_ENREF_46) | | 77% [^32^](#_ENREF_32)^,^[^33^](#_ENREF_33)^,^[^50-52^](#_ENREF_50) | | | 60% [^53^](#_ENREF_53) | | | 44% [^54-56^](#_ENREF_54) | | 70% [^57-61^](#_ENREF_57) | | |  |  |
| Edible crop uptake | | 69% [^10^](#_ENREF_10)^,^[^49^](#_ENREF_49) | | 70% [^10^](#_ENREF_10)^,^[^49^](#_ENREF_49) | | | 67% [^10^](#_ENREF_10)^,^[^53^](#_ENREF_53) | | | 55% [^10^](#_ENREF_10)^,^[^56^](#_ENREF_56) | | 15% [^10^](#_ENREF_10)^,^[^57-61^](#_ENREF_57) | | |  |  |
| Crop Residual Recycled | | 24% [^14^](#_ENREF_14)^,^[^62-66^](#_ENREF_62) | | 21% [^14^](#_ENREF_14)^,^[^62-66^](#_ENREF_62) | | | 25% [^14^](#_ENREF_14)^,^[^62-66^](#_ENREF_62) | | | 25% [^14^](#_ENREF_14)^,^[^62-66^](#_ENREF_62) | | 85% [^14^](#_ENREF_14)^,^[^62-66^](#_ENREF_62) | | |  |  |
| Processing waste | | 82% [^67^](#_ENREF_67) | | 86% [^67^](#_ENREF_67) | | | 55% [^67^](#_ENREF_67) | | | 70% [^67^](#_ENREF_67) | | 70% [^67^](#_ENREF_67) | | |  |  |
| Processing waste Recycled | | 10% [^68-70^](#_ENREF_68) | | 10% [^68-70^](#_ENREF_68) | | | 10% [^68-70^](#_ENREF_68) | | | 10% [^68-70^](#_ENREF_68) | | 10% [^68-70^](#_ENREF_68) | | |  |  |
| Distribution & Eaten | | 95% [^67^](#_ENREF_67) | | 72% [^67^](#_ENREF_67) | | | 65% [^67^](#_ENREF_67) | | | 63% [^67^](#_ENREF_67) | | 63% [^67^](#_ENREF_67) | | |  |  |
| VNF | | 1.2 | | 1.8 | | | 4.9 | | | 8.0 | | 9.4 | | |  |  |
|  | | | Seafood | | | |  | | | |  | | |  | |  |
|  | | | Wild-caught | | | Farmed | Poultry | | | | Egg | | | Dairy products | |  |
| Whole crop uptake | | | 100% [^71-74^](#_ENREF_71) | | | 88% [^32^](#_ENREF_32)^,^[^46^](#_ENREF_46)^,^[^75-78^](#_ENREF_75) | 75% [^32^](#_ENREF_32)^,^[^46^](#_ENREF_46)^,^[^79^](#_ENREF_79)^,^[^80^](#_ENREF_80) | | | | 74% [^32^](#_ENREF_32)^,^[^46^](#_ENREF_46)^,^[^81-83^](#_ENREF_81) | | | 83% [^32^](#_ENREF_32)^,^[^46^](#_ENREF_46)^,^[^84-87^](#_ENREF_84) | |  |
| Edible crop uptake | | | 100% [^71-74^](#_ENREF_71) | | | 84% [^10^](#_ENREF_10)^,^[^49^](#_ENREF_49) | 67% [^10^](#_ENREF_10)^,^[^49^](#_ENREF_49) | | | | 63% [^10^](#_ENREF_10)^,^[^49^](#_ENREF_49) | | | 66% [^10^](#_ENREF_10)^,^[^49^](#_ENREF_49) | |  |
| Crop Residual Recycled | | | 0% [^14^](#_ENREF_14)^,^[^62-66^](#_ENREF_62) | | | 14% [^14^](#_ENREF_14)^,^[^62-66^](#_ENREF_62) | 20% [^14^](#_ENREF_14)^,^[^62-66^](#_ENREF_62) | | | | 19% [^14^](#_ENREF_14)^,^[^62-66^](#_ENREF_62) | | | 22% [^14^](#_ENREF_14)^,^[^62-66^](#_ENREF_62) | |  |
| Animal retain | | | 100% [^71-74^](#_ENREF_71) | | | 37% [^71^](#_ENREF_71)^,^[^74-78^](#_ENREF_74)^,^[^88-92^](#_ENREF_88) | 47% [^61^](#_ENREF_61)^,^ [^62^](#_ENREF_62)^,^ [^75-77^](#_ENREF_75) | | | | 45% [^30^](#_ENREF_30)^,^[^81-83^](#_ENREF_81)^,^[^93^](#_ENREF_93) | | | 35% [^28^](#_ENREF_28)^,^[^81^](#_ENREF_81)^,^[^86^](#_ENREF_86)^,^[^87^](#_ENREF_87)^,^[^94-98^](#_ENREF_94) | |  |
| Animal Waste Recycled | | | 0% [^71-74^](#_ENREF_71) | | | 35% [^71^](#_ENREF_71)^,^[^74^](#_ENREF_74)^,^[^76^](#_ENREF_76)^,^[^78^](#_ENREF_78)^,^[^88^](#_ENREF_88)^,^[^89^](#_ENREF_89) | 36% [^61^](#_ENREF_61)^,^ [^62^](#_ENREF_62)^,^ [^75-77^](#_ENREF_75) | | | | 36% [^63-65^](#_ENREF_63)^,^ [^78^](#_ENREF_78)^,^ [^79^](#_ENREF_79) | | | 13% [^28^](#_ENREF_28)^,^[^81^](#_ENREF_81)^,^[^86^](#_ENREF_86)^,^[^87^](#_ENREF_87)^,^[^94-98^](#_ENREF_94) | |  |
| Carcass retain | | | 86% [^71^](#_ENREF_71)^,^[^74^](#_ENREF_74)^,^[^76^](#_ENREF_76)^,^[^78^](#_ENREF_78)^,^[^88^](#_ENREF_88)^,^[^89^](#_ENREF_89) | | | 86% [^71^](#_ENREF_71)^,^[^74^](#_ENREF_74)^,^[^76^](#_ENREF_76)^,^[^78^](#_ENREF_78)^,^[^88^](#_ENREF_88)^,^[^89^](#_ENREF_89) | 71% [^31^](#_ENREF_31)^,^[^79^](#_ENREF_79)^,^[^80^](#_ENREF_80)^,^[^99^](#_ENREF_99)^,^[^100^](#_ENREF_100) | | | | 96%[^30^](#_ENREF_30)^,^[^81-83^](#_ENREF_81)^,^[^93^](#_ENREF_93) | | | 96% [^63^](#_ENREF_63)^,^ [^68^](#_ENREF_68)^,^ [^69^](#_ENREF_69)^,^ [^77-82^](#_ENREF_77) | |  |
| Carcass waste Recycled | | | 85% [^101^](#_ENREF_101) | | | 85% [^101^](#_ENREF_101) | 85% [^101^](#_ENREF_101) | | | | 85% [^101^](#_ENREF_101) | | | 85% [^101^](#_ENREF_101) | |  |
| Processing waste | | | 94% [^67^](#_ENREF_67) | | | 94% [^67^](#_ENREF_67) | 89% [^67^](#_ENREF_67) | | | | 100% [^67^](#_ENREF_67) | | | 99% [^67^](#_ENREF_67) | |  |
| Processing waste Recycled | | | 10% [^68-70^](#_ENREF_68) | | | 10% [^68-70^](#_ENREF_68) | 10% [^68-70^](#_ENREF_68) | | | | 10% [^68-70^](#_ENREF_68) | | | 10% [^68-70^](#_ENREF_68) | |  |
| Distribution & Eaten | | | 68% [^67^](#_ENREF_67) | | | 68% [^67^](#_ENREF_67) | 85% [^67^](#_ENREF_67) | | | | 85% [^67^](#_ENREF_67) | | | 85% [^67^](#_ENREF_67) | |  |
| VNF | | | 0.6 | | | 4.2 | 4.8 | | | | 4.0 | | | 4.6 | |  |
|  | | |  | | |  |  | | | |  | | |  | |  |
|  | | |  | | |  |  | | | |  | | |  | |  |
|  | | |  | | |  |  | | | |  | | |  | |  |
|  | |  |  | | | | | | |  | | | | | |  |
|  | | |  | Lamb | | | | |  | | | Beef | | |  | |
|  | | | Pork | Grazing | | | | | Feedlot | | | Grazing | | | Feedlot | |
| Whole crop uptake | | | 74% [^27^](#_ENREF_27)^,^[^32^](#_ENREF_32)^,^[^46^](#_ENREF_46)^,^[^102^](#_ENREF_102)^,^[^103^](#_ENREF_103) | 100% [^52^](#_ENREF_52)^,^[^85^](#_ENREF_85)^,^[^104-106^](#_ENREF_104) | | | | | 81% [^32^](#_ENREF_32)^,^[^46^](#_ENREF_46)^,^[^107^](#_ENREF_107)^,^[^108^](#_ENREF_108) | | | 100% [^52^](#_ENREF_52)^,^[^85^](#_ENREF_85)^,^[^104-106^](#_ENREF_104) | | | 77% [^32^](#_ENREF_32)^,^[^46^](#_ENREF_46)^,^[^109-111^](#_ENREF_109) | |
| Edible crop uptake | | | 66% [^10^](#_ENREF_10)^,^[^49^](#_ENREF_49) | 99% [^10^](#_ENREF_10)^,^[^49^](#_ENREF_49) | | | | | 68% [^10^](#_ENREF_10)^,^[^49^](#_ENREF_49) | | | 99% [^27^](#_ENREF_27)^,^ [^42^](#_ENREF_42) | | | 67% [^10^](#_ENREF_10)^,^[^49^](#_ENREF_49) | |
| Crop Residual Recycled | | | 19% [^14^](#_ENREF_14)^,^[^62-66^](#_ENREF_62) | 1% [^14^](#_ENREF_14)^,^[^62-66^](#_ENREF_62) | | | | | 23% [^14^](#_ENREF_14)^,^[^62-66^](#_ENREF_62) | | | 1% [^14^](#_ENREF_14)^,^[^62-66^](#_ENREF_62) | | | 22% [^14^](#_ENREF_14)^,^[^62-66^](#_ENREF_62) | |
| Animal retain | | | 44% [^27^](#_ENREF_27)^,^[^102^](#_ENREF_102)^,^[^103^](#_ENREF_103)^,^[^112-114^](#_ENREF_112) | 12% [^26^](#_ENREF_26)^,^[^94^](#_ENREF_94)^,^[^108^](#_ENREF_108)^,^[^115-121^](#_ENREF_115) | | | | | 14% [^26^](#_ENREF_26)^,^[^94^](#_ENREF_94)^,^[^107^](#_ENREF_107)^,^[^115-117^](#_ENREF_115)^,^[^120^](#_ENREF_120)^,^[^122^](#_ENREF_122) | | | 7% [^109-111^](#_ENREF_109)^,^[^122-127^](#_ENREF_122) | | | 14% [^109-111^](#_ENREF_109)^,^[^122-125^](#_ENREF_122)^,^[^127-132^](#_ENREF_127) | |
| Animal Waste Recycled | | | 11% [^87-89^](#_ENREF_87)^,^ [^98-100^](#_ENREF_98) | 79% [^26^](#_ENREF_26)^,^[^94^](#_ENREF_94)^,^[^108^](#_ENREF_108)^,^[^115^](#_ENREF_115)^,^[^116^](#_ENREF_116)^,^[^119^](#_ENREF_119)^,^[^120^](#_ENREF_120)^,^[^122^](#_ENREF_122) 15% [^26^](#_ENREF_26)^,^[^94^](#_ENREF_94)^,^[^108^](#_ENREF_108)^,^[^115^](#_ENREF_115)^,^[^116^](#_ENREF_116)^,^[^119^](#_ENREF_119)^,^[^120^](#_ENREF_120)^,^[^122^](#_ENREF_122) | | | | | | | | 79% [^109-111^](#_ENREF_109)^,^[^122-132^](#_ENREF_122) 15% [^95-97^](#_ENREF_95)^,^ [^109-119^](#_ENREF_109) | | | | |
| Carcass retain | | | 76% [^27^](#_ENREF_27)^,^[^102^](#_ENREF_102)^,^[^103^](#_ENREF_103)^,^[^112-114^](#_ENREF_112) | 47% [^26^](#_ENREF_26)^,^[^94^](#_ENREF_94)^,^[^107^](#_ENREF_107)^,^[^108^](#_ENREF_108)^,^[^115-122^](#_ENREF_115) 47% [^26^](#_ENREF_26)^,^[^94^](#_ENREF_94)^,^[^107^](#_ENREF_107)^,^[^108^](#_ENREF_108)^,^[^115-122^](#_ENREF_115) | | | | | | | | 55% [^109-111^](#_ENREF_109)^,^[^122-132^](#_ENREF_122) 55% [^109-111^](#_ENREF_109)^,^[^122-132^](#_ENREF_122) | | | | |
| Carcass waste Recycled | | | 85% [^101^](#_ENREF_101) | 85% [^101^](#_ENREF_101) | | | | | 85% [^101^](#_ENREF_101) | | | 85% [^101^](#_ENREF_101) | | | 85% [^101^](#_ENREF_101) | |
| Processing waste | | | 92% [^67^](#_ENREF_67) | 83% [^67^](#_ENREF_67) | | | | | 83% [^67^](#_ENREF_67) | | | 92% [^67^](#_ENREF_67) | | | 92% [^67^](#_ENREF_67) | |
| Processing waste Recycled | | | 10% [^68-70^](#_ENREF_68) | 10% [^68-70^](#_ENREF_68) | | | | | 10% [^68-70^](#_ENREF_68) | | | 10% [^68-70^](#_ENREF_68) | | | 10% [^68-70^](#_ENREF_68) | |
| Distribution & Eaten | | | 85% [^67^](#_ENREF_67) | 85% [^67^](#_ENREF_67) | | | | | 85% [^67^](#_ENREF_67) | | | 85% [^67^](#_ENREF_67) | | | 85% [^67^](#_ENREF_67) | |
| VNF | | | 5.5 | 5.7 | | | | | 29.8 | | | 7.4 | | | 25.2 | |


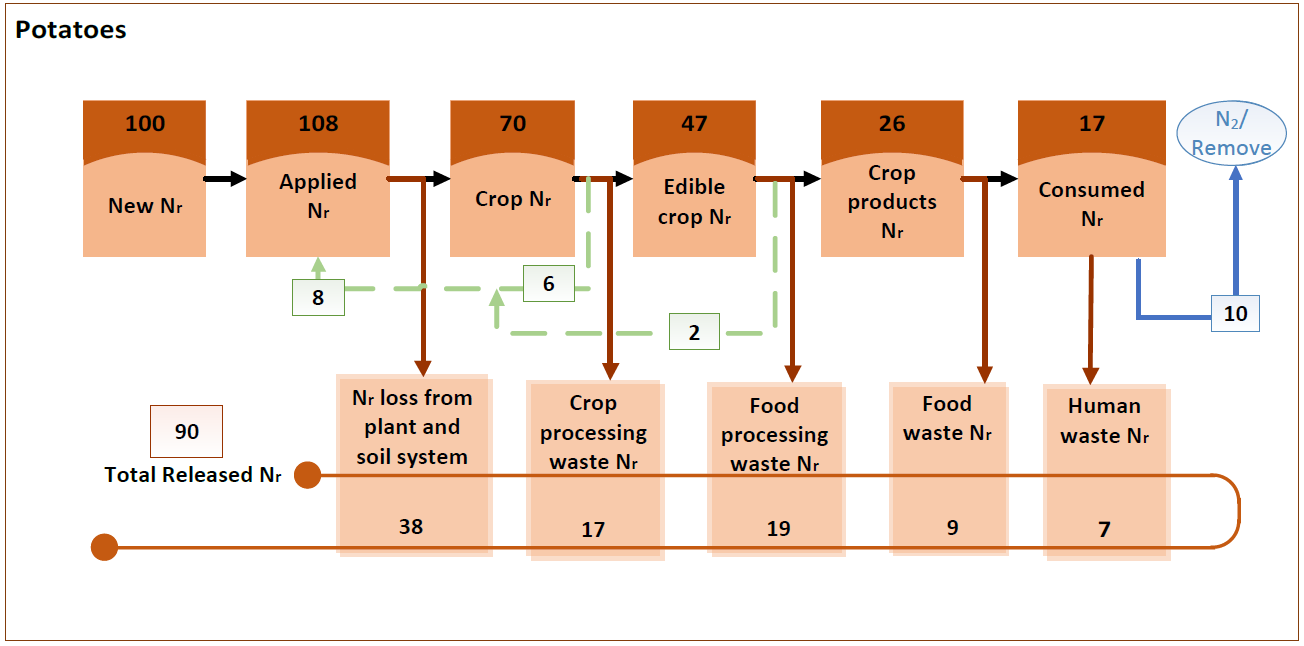
**
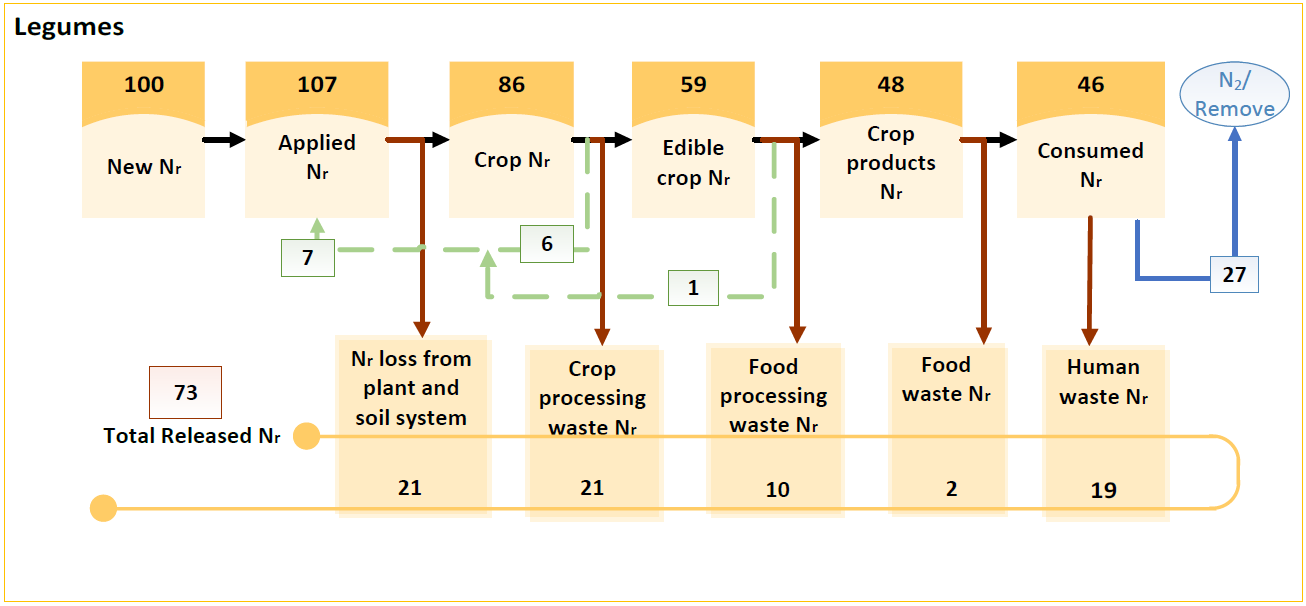

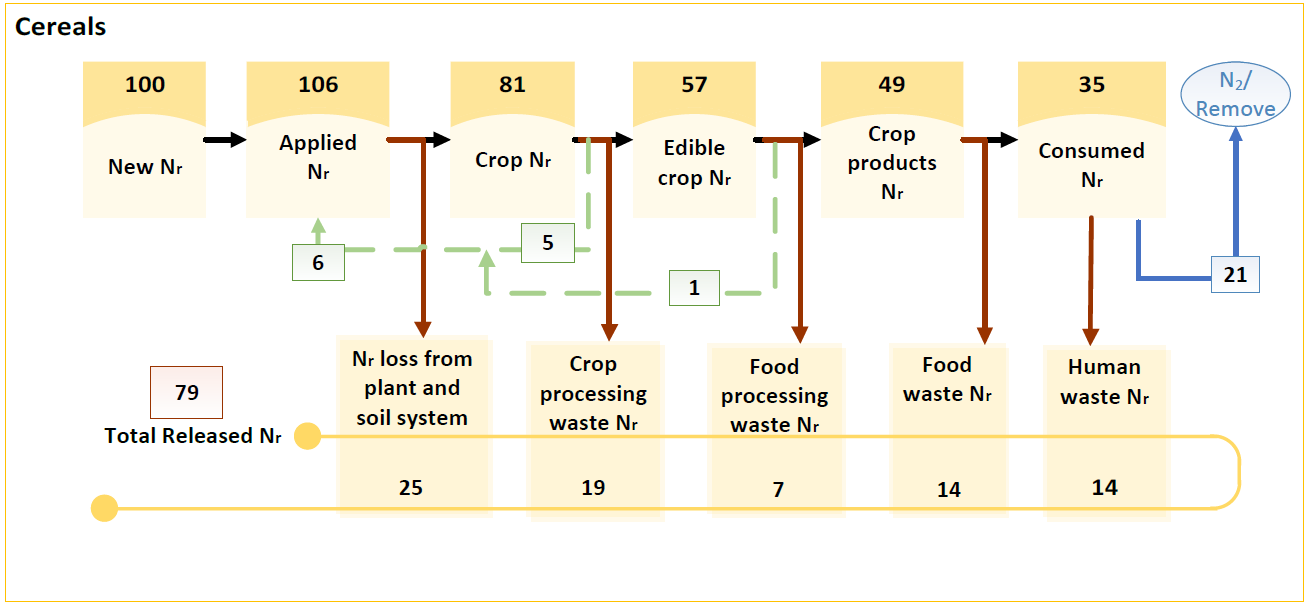
Supplementary Fig 1: Nr flow along the entire production and consumption chain of 11 major food categories in Australia.**

**
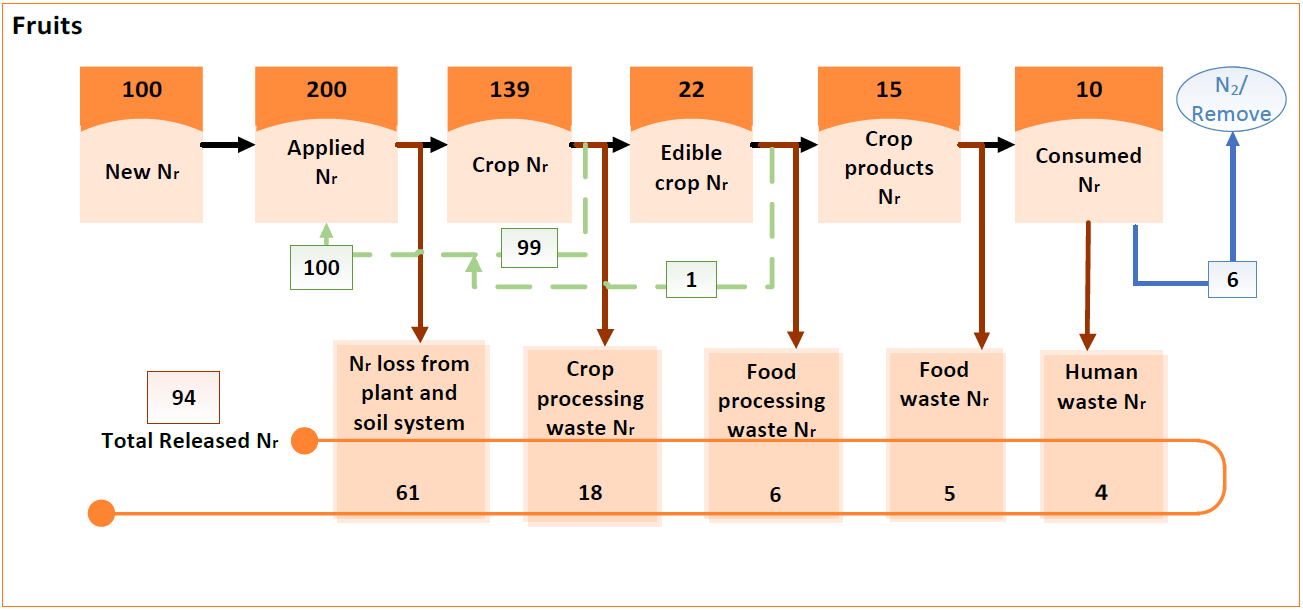

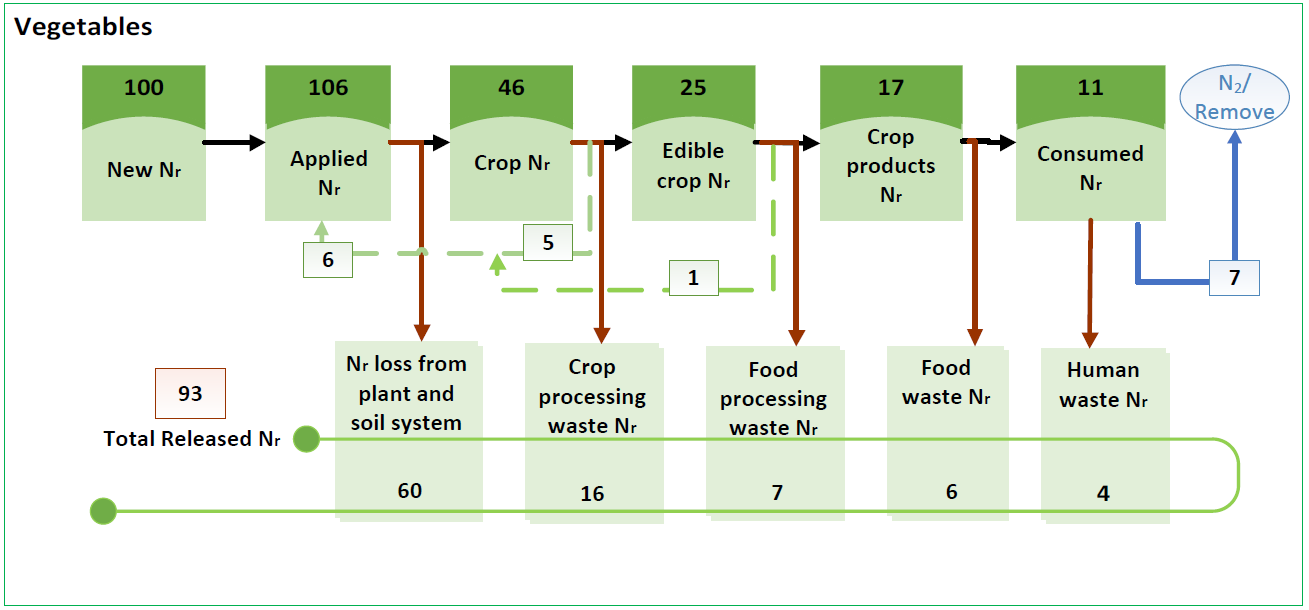
**


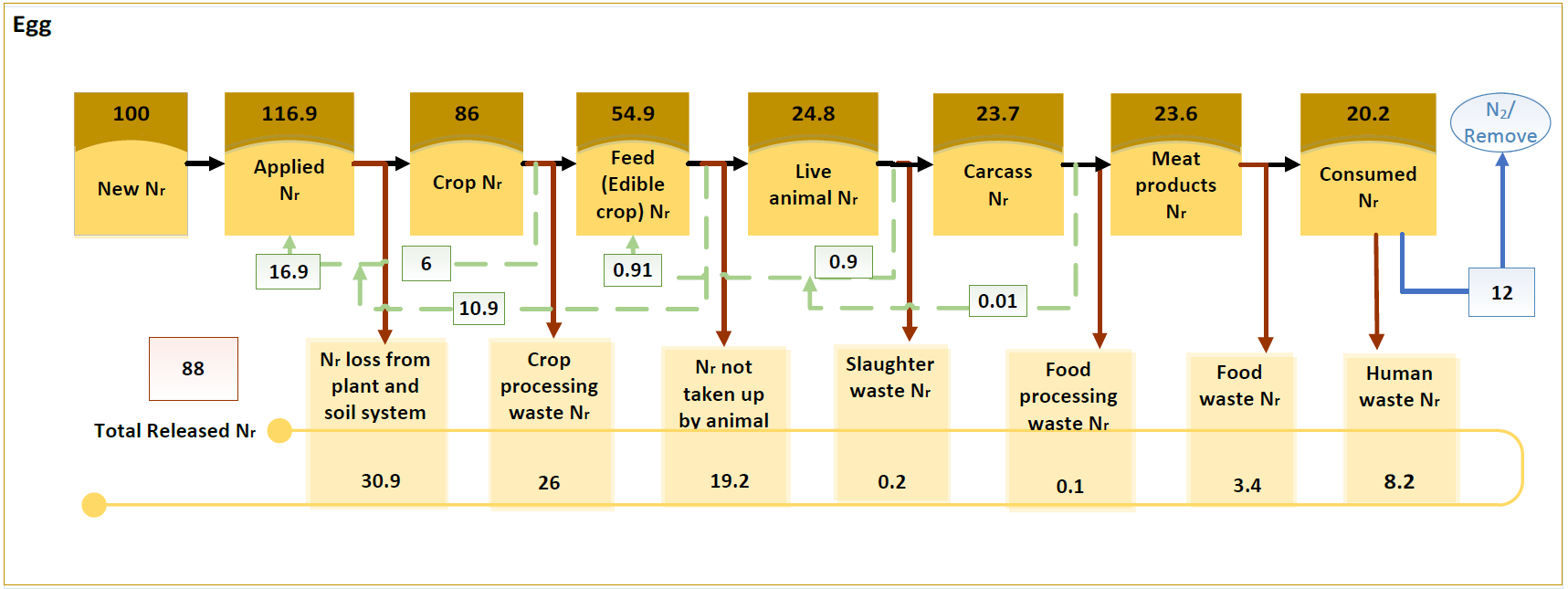


**
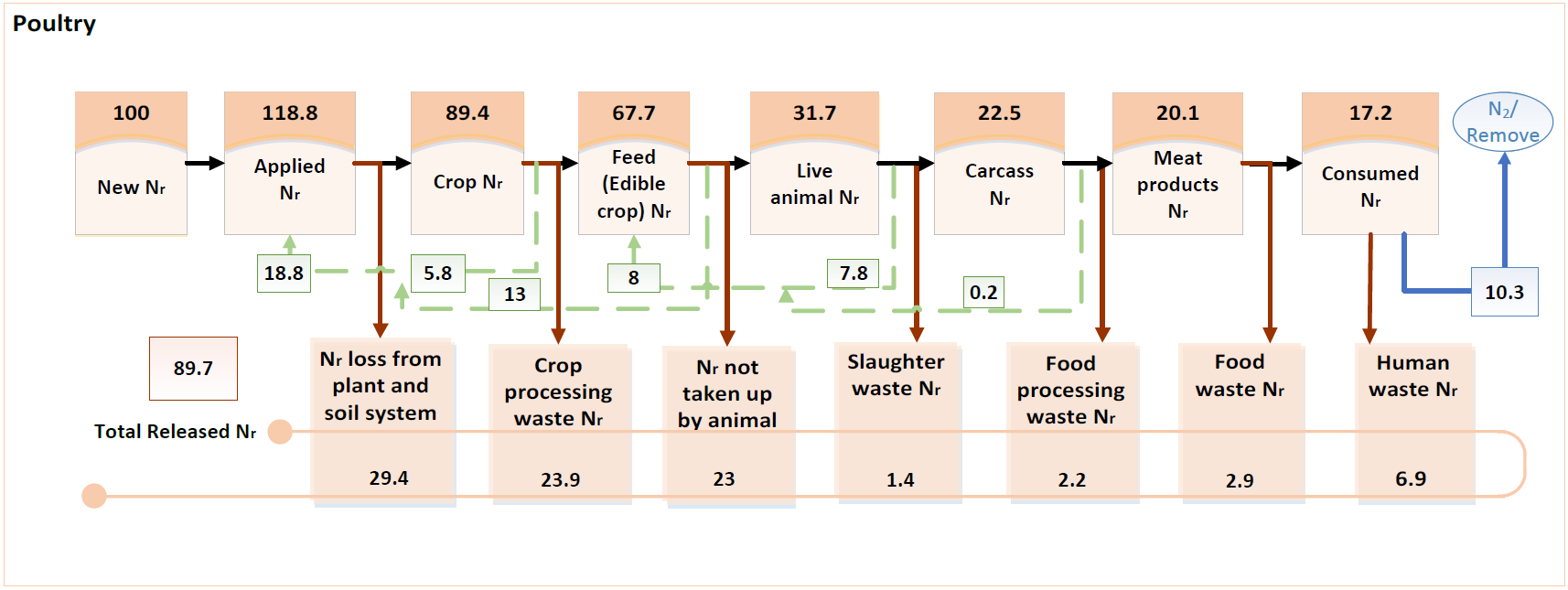
**
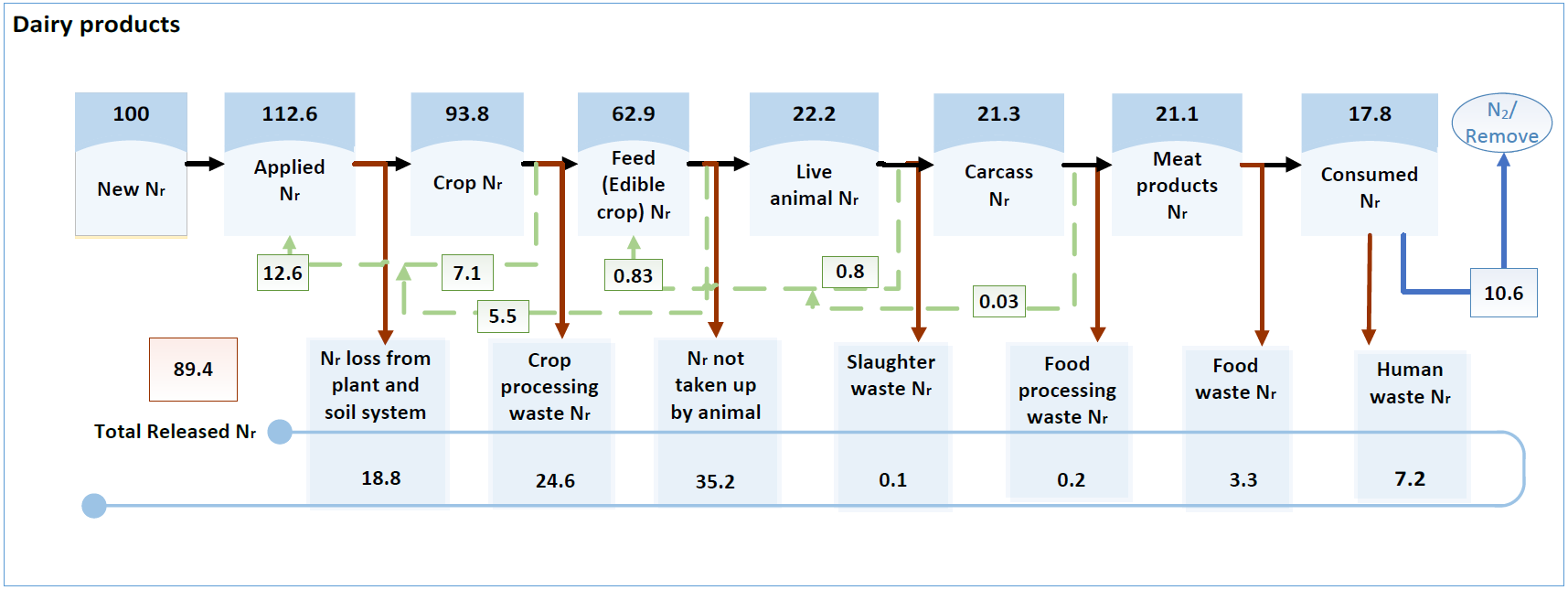


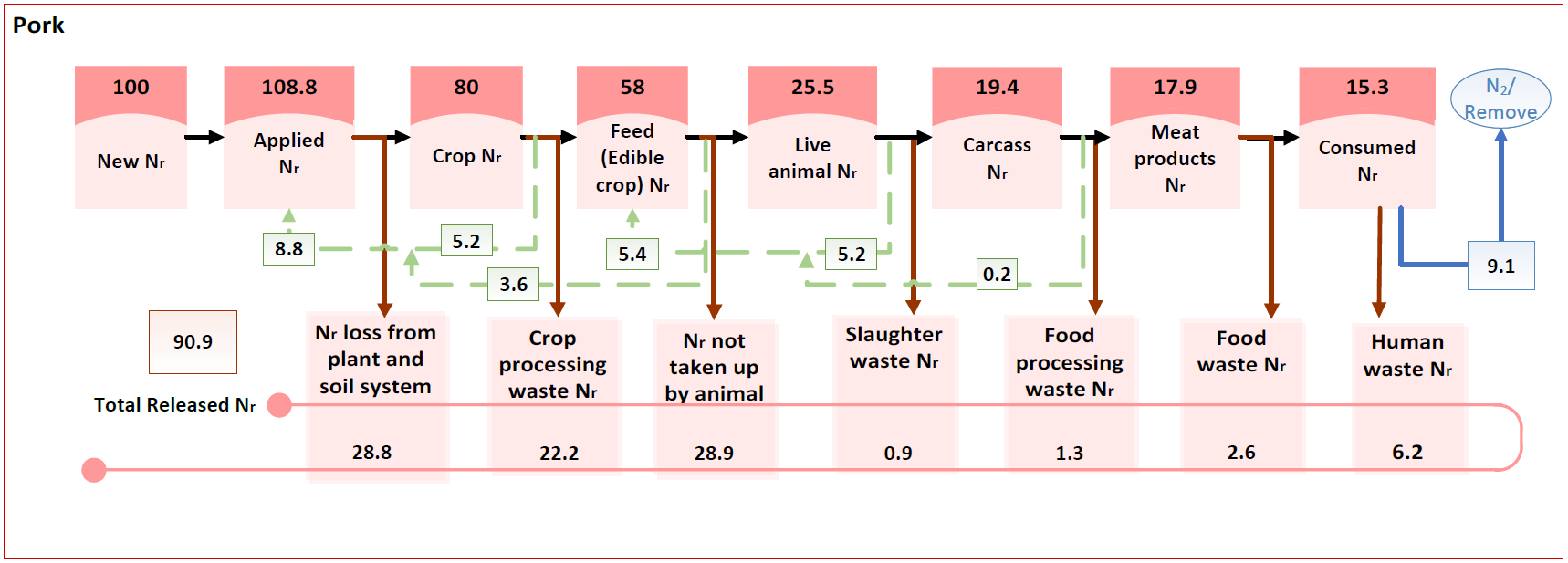


**
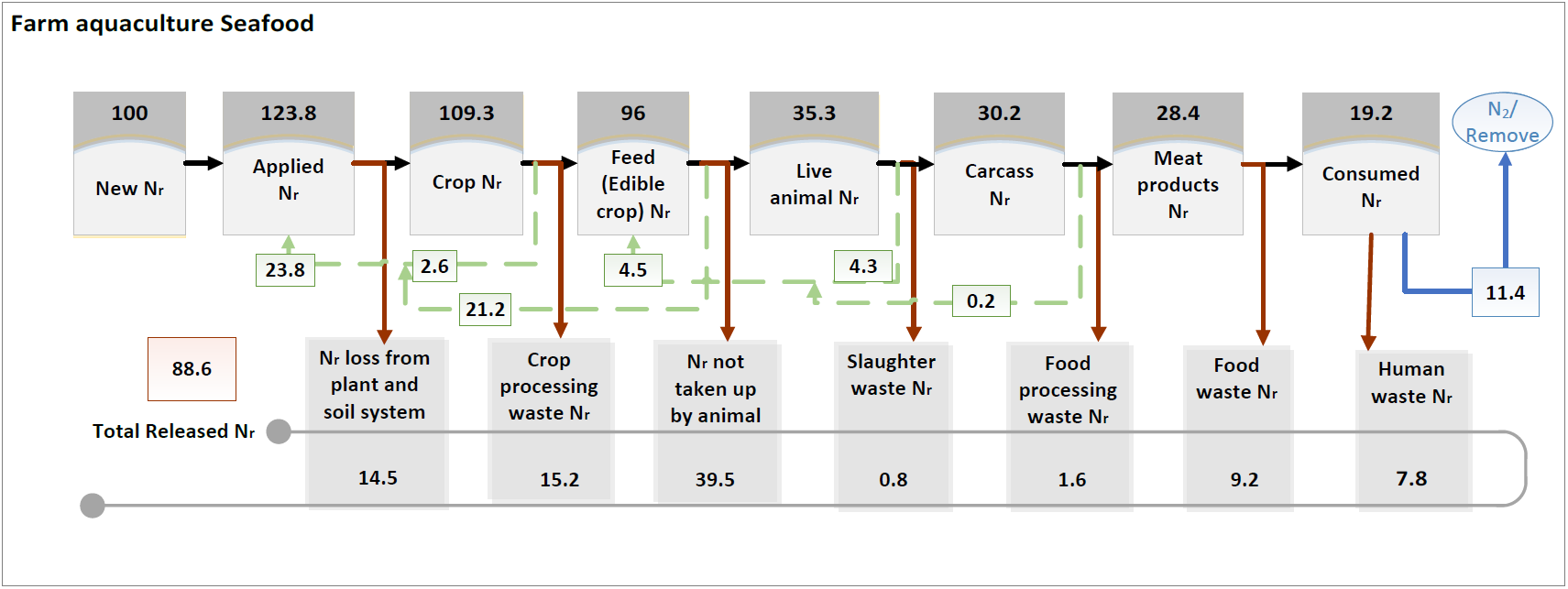

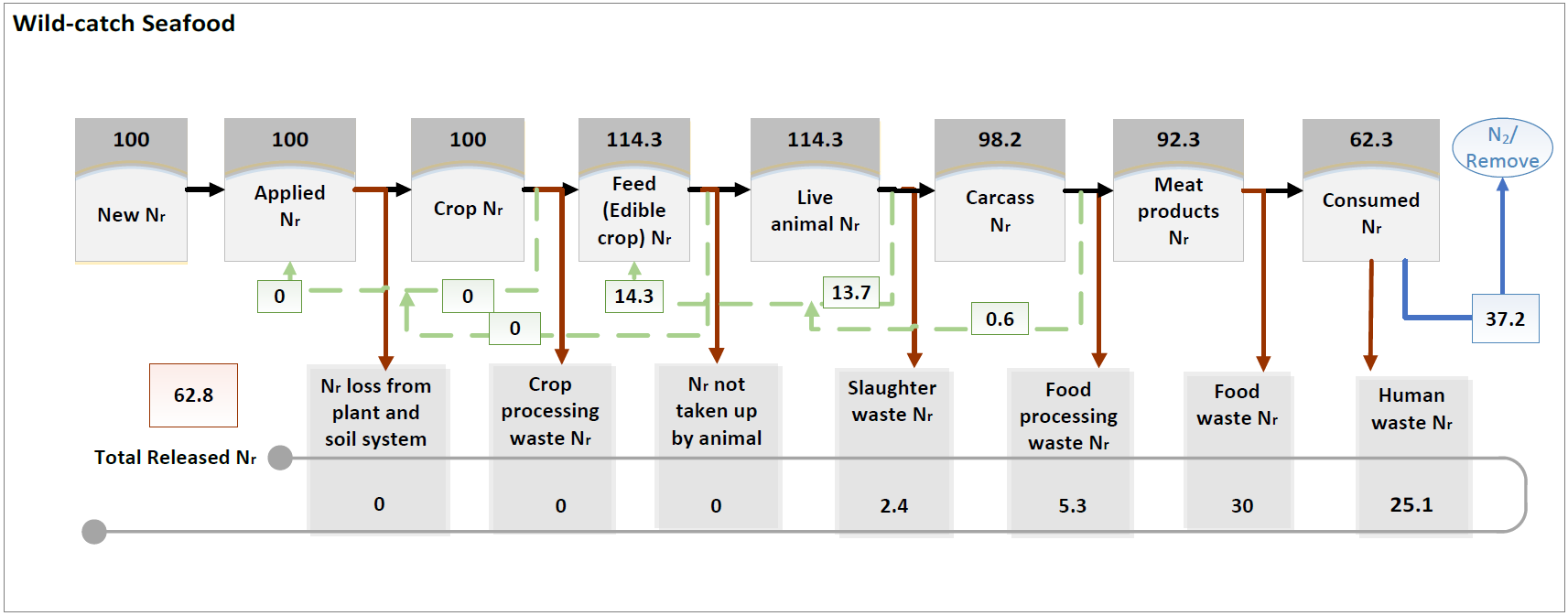
**

**
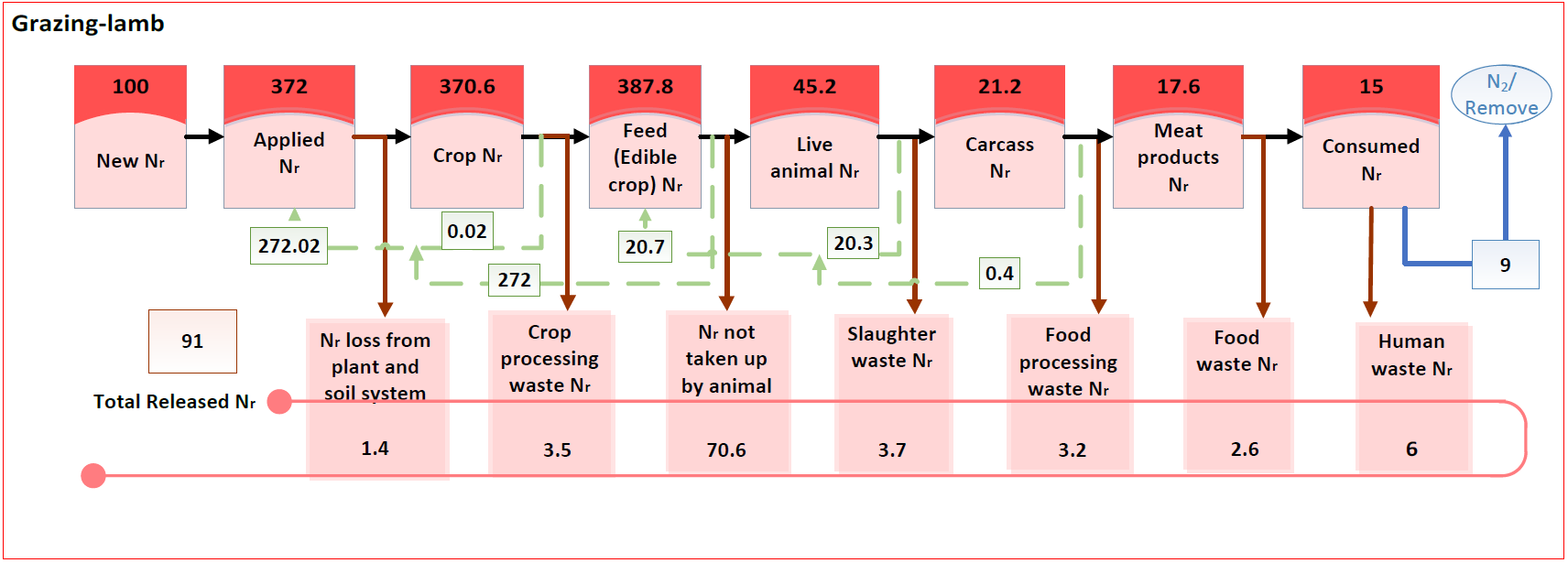
**


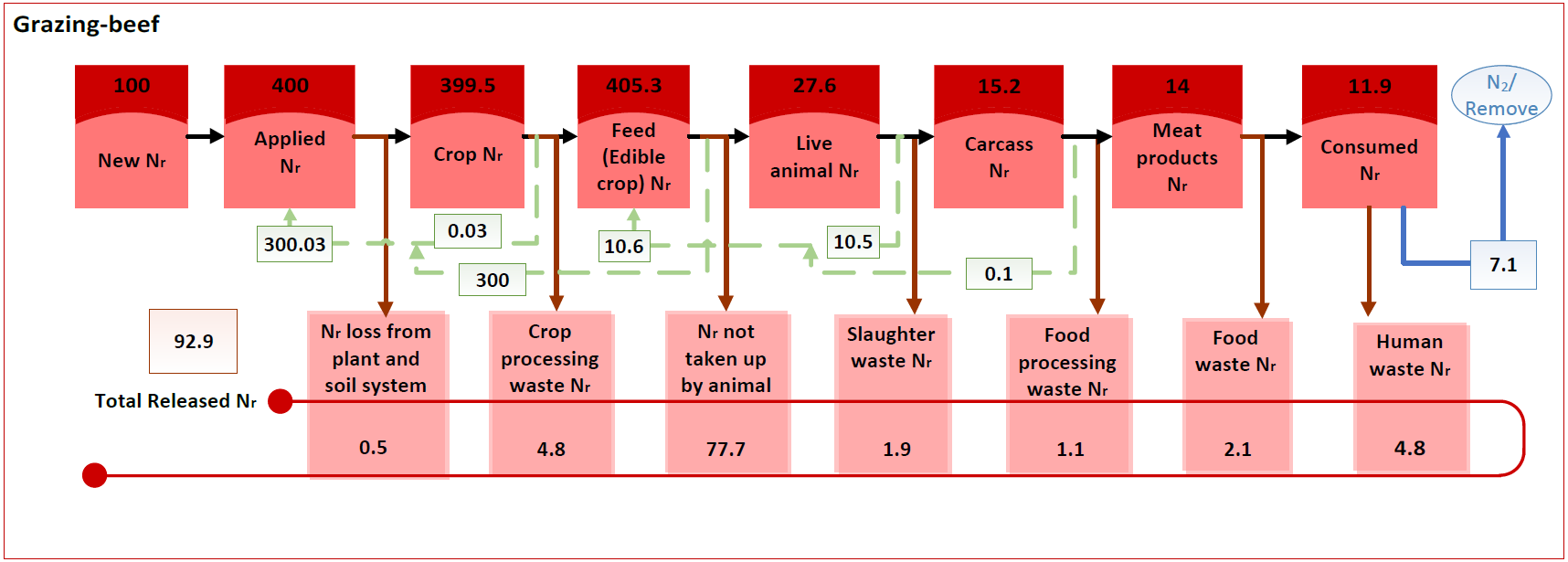

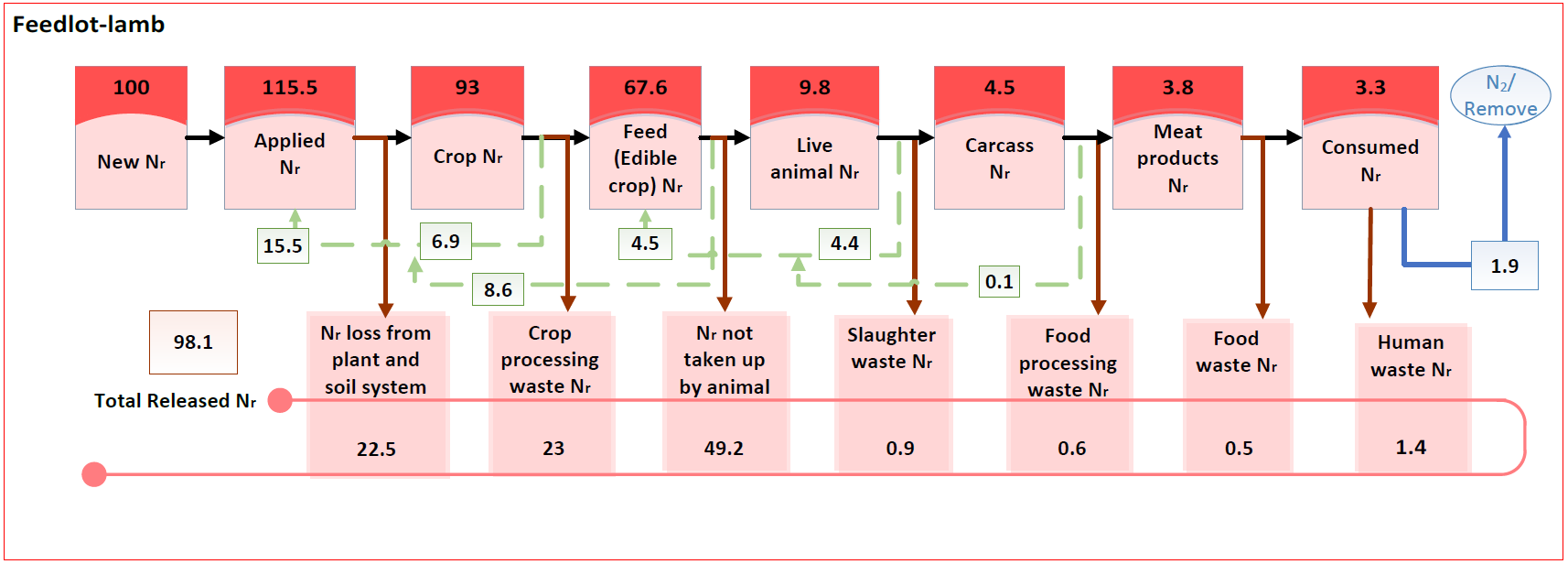


**
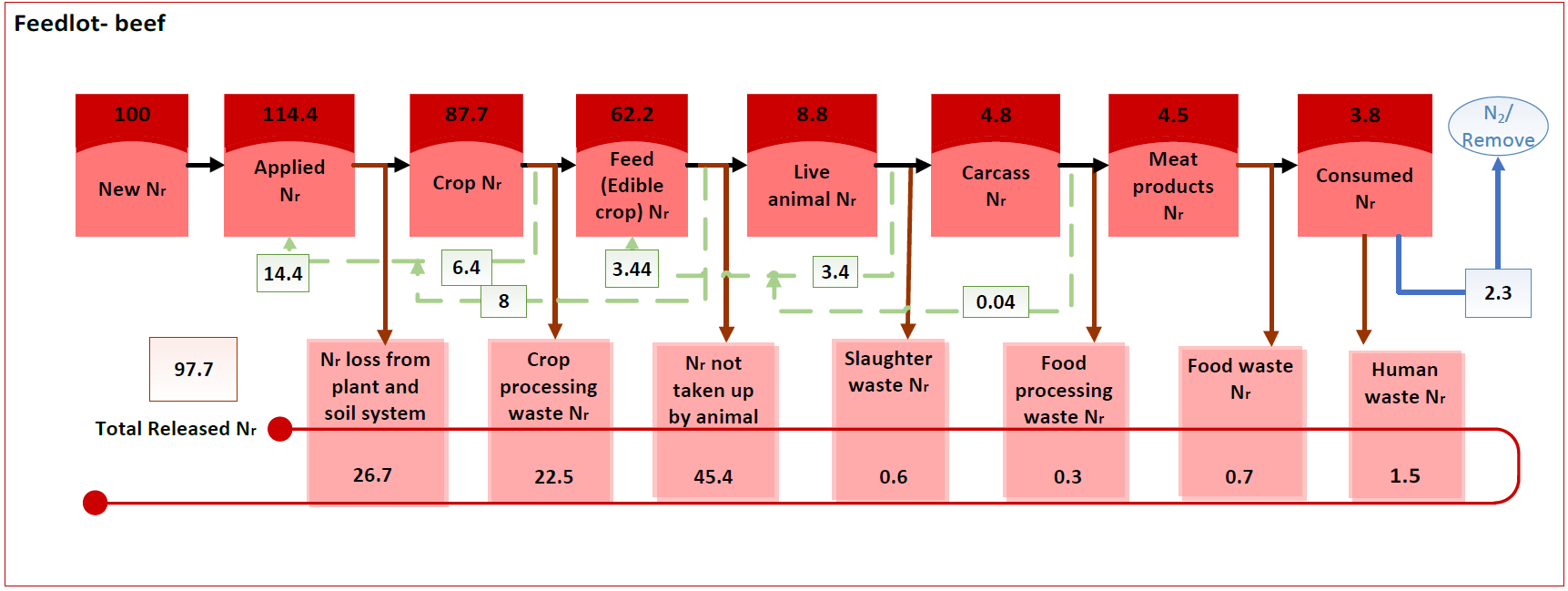
**

**References**

1 Leach, A. M. *et al.* A nitrogen footprint model to help consumers understand their role in nitrogen losses to the environment. *Environmental Development*. **1**, 40-66 (2012).

2 Stevens, C. J., Leach, A. M., Dale, S. & Galloway, J. N. Personal nitrogen footprint tool for the United Kingdom. *Environmental Science: Processes & Impacts*. **16**, 1563-1569 (2014).

3 Gonçalves, V. M. P. *Impact of nitrogen into the environment. A step on nitrogen footprint calculation in Lisbon, Portugal*, (in review).

4 Pierer, M., Winiwarter, W., Leach, A. M. & Galloway, J. N. The nitrogen footprint of food products and general consumption patterns in Austria. *Food Policy*. **49**, 128-136 (2014).

5 Shibata, H., Cattaneo, L. R., Leach, A. M. & Galloway, J. N. First approach to the Japanese nitrogen footprint model to predict the loss of nitrogen to the environment. *Environmental Research Letters*. **9**, 115013 (2014).

6 Hutton, O. *et al.* Toward a nitrogen footprint calculator for Tanzania. *Environ. Res. Lett* (in review).

7 FAOSTAT. *Food and Agricultural Organization of the United Nations Statistical Database, Food Balance Sheet* <http://faostat3.fao.org/download/FB/CC/E> (2016) (Date of access: 5/12/2015).

8 Heffer, P. Assessment of fertilizer use by crop at the global level. *International Fertilizer Industry Association, Paris, Assessment of Fertilizer Use by Crop at the Global Level 2010-2010, 11.* (2013).

9 Gustavsson, J., Cederberg, C., Sonesson, U. & Emanuelsson, A. The methodology of the FAO study:“Global Food Losses and Food Waste–extent, causes and prevention”–FAO, 2011. *The Swedish Institute for Food and Biotechnology (SIK), Göteborg, Sweden* (2013).

10 IPNI. *IPNI Estimates of nutrient uptake and removal.* <http://www.ipni.net/article/IPNI-3296> (2012) (Date of access: 10/2/2015).

11 ABARES. *Australian Bureau of Agricultural and Resource Economics and Sciences* <http://www.agriculture.gov.au/abares/data> (2015) (Date of access: 11/11/2015).

12 ABS. *Australian Bureau of Statistics: Agricultural Resource Management Practices, 4630.0* <http://www.abs.gov.au/AUSSTATS/abs@.nsf/DetailsPage/4630.02011-12?OpenDocument> (2015) (Date of access: 15/10/2015).

13 ABS. *Australian Bureau of Statistics: Australian Environmental-Economic Accounts, 4655.0* <http://www.abs.gov.au/AUSSTATS/abs@.nsf/DetailsPage/4655.02015?OpenDocument> (2015) (Date of access: 19/12/2015).

14 ABS. *Australian Bureau of Statistics: Land Management Practices Survey (LaMPS)* <http://stat.abs.gov.au/Index.aspx?DataSetCode=ABS_LAMPS> (2013) (Date of access: 15/4/2016).

15 NHMRC. Australian Dietary Guidelines. (*National Health and Medical Research Council*) <https://www.nhmrc.gov.au/guidelines-publications/n55> (2013) (Date of access: 1/4/2016).

16 BREE. Australian energy projections to 2034–35. (*Bureau of Resources and Energy Economics, Canberra, Australia.*) <http://www.industry.gov.au/Office-of-the-Chief-Economist/Publications/Documents/aep/australianenergyprojections2034-35report.docx> (2011) (Date of access: 25/3/2015).

17 COAG. National Strategy on Energy Efficiency. (*Council of Australian Governments*) <https://www.coag.gov.au/sites/default/files/nsee_update_july_2010.pdf> (2010) (Date of access: 9/8/2015).

18 DIRD. *Vehicle Emissions Standards* <https://infrastructure.gov.au/roads/environment/emission/> (2015) (Date of access: 15/10/2015).

19 DIS. Energy White Paper 2015. (*Department of Industry and Science, Government of Australia*) <http://www.industry.gov.au/EnergyWhitePaperataglance/files/Energy-White-Paper-2015.pdf> (2015) (Date of access: 13/8/2015).

20 AAA. Motoring Report 2013. (*Australian Automobile Association*) <http://www.aaa.asn.au/storage/AAA%20Motoring%20Report%202013.pdf> (2013) (Date of access: 10/4/2015).

21 DIRD. Vehicle Emissions Discussion Paper. (*The Department of Infrastructure and Regional Development*) <https://infrastructure.gov.au/roads/environment/forum/files/Vehicle_Emissions_Discussion_Paper.pdf> (2016) (Date of access: 15/4/2015).

22 CCA. Reducing Australia’s Greenhouse Gas Emissions—Targets and Progress Review: Final Report. (*Climate Change Authority, Australian Government*) <http://climatechangeauthority.gov.au/files/files/Target-Progress-Review/Targets%20and%20Progress%20Review%20Final%20Report.pdf> (2014) (Date of access: 1/12/2015).

23 DCCEE. Transport Emissions Projections, Department of Climate Change and Energy Efficiency. Canberra: Commonwealth of Australia. <http://climatechangeauthority.gov.au/publications> (2012) (Date of access: 15/4/2015).

24 DoE. National Inventory Report 2012 Volume 1. (*Australian National Greenhouse Accounts, Australia.*) <https://www.environment.gov.au/system/files/resources/6b894230-f15f-4a69-a50c-5577fecc8bc2/files/national-inventory-report-2012-vol1.pdf> (2014b) (Date of access: 16/2/2015).

25 WSAA. The Australian urban water industry : WSAA facts. (*Water Services Association of Australia*) <http://www.worldcat.org/title/australian-urban-water-industry-wsaa-facts/oclc/57334333> (2005) (Date of access: 15/12/2015).

26 MLA. *Meat and Livestock Australia* <http://www.Dairyaustralia.com.au> (2015) (Date of access: 15/9/2015).

27 APL. *Australian Pork Limited* <http://www.Dairyaustralia.com.au> (2015) (Date of access: 19/11/2015).

28 DA. *Dairy Australia* <http://www.Dairyaustralia.com.au> (2015) (Date of access: 22/10/2015).

29 NSIA. *National Seafood Industry Alliance* <http://www.seafoodforaustralia.com.au> (2015) (Date of access: 9/9/2015).

30 AECL. *Australian Egg Corporation Limited* <https://www.aecl.org> (2015) (Date of access: 15/10/2015).

31 ACMF. *Australian Chicken Meat Federation* <http://www.chicken.org.au> (2015) (Date of access: 6/9/2015).

32 Chen, D. *et al.* Prospects of improving efficiency of fertiliser nitrogen in Australian agriculture: a review of enhanced efficiency fertilisers. *Soil Research*. **46**, 289-301 (2008).

33 Lam, S. K., Chen, D., Norton, R. & Armstrong, R. Nitrogen demand and the recovery of 15N-labelled fertilizer in wheat grown under elevated carbon dioxide in southern Australia. *Nutrient Cycling in Agroecosystems*. **92**, 133-144, doi:10.1007/s10705-011-9477-6 (2012).

34 WNA. World Nuclear Association: Australia's Electricity. <http://www.world-nuclear.org/information-library/country-profiles/countries-a-f/appendices/australia-s-electricity.aspx> (2015) (Date of access: 20/5/2016).

35 Brander, A. M., Sood, A., Wylie, C., Haughton, A., Lovell, J., Reviewers, I., & Davis, G. . *Electricity-specific emission factors for grid electricity* <https://ecometrica.com/assets/Electricity-specific-emission-factors-for-grid-electricity.pdf> (2011) (Date of access: 20/2/2015).

36 Elliston, B., Diesendorf, M. & MacGill, I. Simulations of scenarios with 100% renewable electricity in the Australian National Electricity Market. *Energy Policy*. **45**, 606-613 (2012).

37 Combet, G. National Greenhouse and Energy Reporting (Measurement) Amendment Determination 2013 (No. 1) (Federal Register of Legislation) <https://www.legislation.gov.au/Details/F2013L01191> (2015) (Date of access: 30/11/2015).

38 ABS. *Australian Bureau of Statistics: Australia’s Demographic Statistics -3101.0* <http://www.abs.gov.au/ausstats/abs@.nsf/0/BCDDE4F49C8A3D1ECA257B8F00126F77?Opendocument> (2016) (Date of access: 5/6/2015).

39 ABS. *Australian Bureau of Statistics: Household Income and Income Distribution-6523.0* <http://www.abs.gov.au/AUSSTATS/abs@.nsf/Lookup/6523.0Main+Features22011-12> (2013) (Date of access: 10/2/2015).

40 BITRE. Australian Infrastructure Statistics Yearbook 2012. (*Department of Infrastructure and Transport, Bureau of Infrastructure, Transport and Regional Economics*) <https://bitre.gov.au/publications/2012/files/stats_002.pdf> (2012) (Date of access: 5/5/2015).

41 BITRE. Australian infrastructure statistics Yearbook 2013. (*Department of Infrastructure and Regional Development, Bureau of Infrastructure, Transport and Regional Economics*) <https://bitre.gov.au/publications/2013/files/INFRA1886_R_BITRE_INFRASTRUCTURE_YEARBOOK_0813_web.pdf> (2013) (Date of access: 15/7/2016).

42 BITRE. Greenhouse gas emissions from Australian transportt: projections to 2020. <https://bitre.gov.au/publications/2009/files/wp_073.pdf> (2009) (Date of access: 10/2/2015).

43 ABS. *Australian Bureau of Statistics: Motor Vehicle Census-9309.0* <http://www.abs.gov.au/ausstats/abs@.nsf/mf/9309.0> (2016) (Date of access: 15/3/2015).

44 ABS. *Australian Bureau of Statistics: Survey of Motor Vehicle Use-9208.0* <http://www.abs.gov.au/ausstats/abs@.nsf/mf/9208.0/> (2014) (Date of access: 23/11/2015).

45 Kitzes, J. An introduction to environmentally-extended input-output analysis. *Resources*. **2**, 489-503 (2013).

46 Herridge, D. F., Peoples, M. B. & Boddey, R. M. Global inputs of biological nitrogen fixation in agricultural systems. *Plant and Soil*. **311**, 1-18, doi:10.1007/s11104-008-9668-3 (2008).

47 Unkovich, M. *et al.* Measuring plant-associated nitrogen fixation in agricultural systems. Report No. 1921531266, (*Australian Centre for International Agricultural Research (ACIAR)*) <http://aciar.gov.au/files/node/10169/mn136_measuring_plant_associated_nitrogen_fixation_19979.pdf> (2008) (Date of access: 17/7/2015).

48 Peoples, M. *et al.* The contributions of nitrogen-fixing crop legumes to the productivity of agricultural systems. *Symbiosis*. **48**, 1-17 (2009).

49 Drew, E. *et al.* Inoculating legumes: a practical guide. (*Grains Research and Development Corporation*) <http://www.agwine.adelaide.edu.au/research/farming/legumes-nitrogen/legume-inoculation/grdc_booklet_inoculating_legumes.pdf> (2012) (Date of access: 10/4/2015).

50 Gardner, J. B. & Drinkwater, L. E. The fate of nitrogen in grain cropping systems: a meta-analysis of 15N field experiments. *Ecological Applications*. **19**, 2167-2184 (2009).

51 Ladha, J. K., Pathak, H., J. Krupnik, T., Six, J. & van Kessel, C. Efficiency of Fertilizer Nitrogen in Cereal Production: Retrospects and Prospects. **87**, 85-156, doi:10.1016/s0065-2113(05)87003-8 (2005).

52 Lester, D. W., Birch, C. J. & Dowling, C. W. Fertiliser N and P application on two Vertosols in north-eastern Australia. 3. Grain N uptake and yield by crop/fallow combination, and cumulative grain N removal and fertiliser N recovery in grain. *Crop and Pasture Science*. **61**, 24-31 (2010).

53 He, Z., Larkin, R. & Honeycutt, W. *Sustainable potato production: global case studies*. (Springer Science & Business Media, 2012).

54 Lam, S. K. *et al.* Measurement and mitigation of nitrous oxide emissions from a high nitrogen input vegetable system. *Scientific reports*. **5** (2015).

55 Cui, M. *et al.* Effective mitigation of nitrate leaching and nitrous oxide emissions in intensive vegetable production systems using a nitrification inhibitor, dicyandiamide. *Journal of Soils and Sediments*. **11**, 722-730 (2011).

56 Harper, S. Environmental effects of vegetable production on sensitive waterways. (2014).

57 Prasertsak, P., Freney, J., Saffigna, P., Denmead, O. & Prove, B. Fate of urea nitrogen applied to a banana crop in the wet tropics of Queensland. *Nutrient Cycling in Agroecosystems*. **59**, 65-73 (2001).

58 Hippler, F. W. R. *et al.* Uptake and Distribution of Soil Applied Zinc by Citrus Trees—Addressing Fertilizer Use Efficiency with 68 Zn Labeling. *PloS one*. **10**, e0116903 (2015).

59 Phogat, V. *et al.* Evaluation of water movement and nitrate dynamics in a lysimeter planted with an orange tree. *Agricultural Water Management*. **127**, 74-84, doi:10.1016/j.agwat.2013.05.017 (2013).

60 Paramasivam, S., Alva, A., Fares, A. & Sajwan, K. Fate of nitrate and bromide in an unsaturated zone of a sandy soil under citrus production. *Journal of Environmental Quality*. **31**, 671-681 (2002).

61 Boman, B. J. & Battikhi, A. M. Growth, evapotranspiration, and nitrogen leaching from young lysimeter-grown orange trees. *Journal of irrigation and drainage engineering*. **133**, 350-358 (2007).

62 DEDJTR. Stubble Burning. (*Agriculture Victoria* )<http://agriculture.vic.gov.au/agriculture/grains-and-other-crops/crop-production/stubble-burning> (2016) (Date of access: 15/2/2015).

63 Jon Midwood, P. B., Andrew Witlock, Matt McCallum. Managing Stubble. (*Australian Government, Grains Research & Development Corporation*) <https://grdc.com.au/~/media/2B5EFD71C2D04212827E2E045E022DE6.pdf>. (2010) (Date of access: 22/4/2015).

64 Scott, B., Podmore, C., Burns, H., Bowden, P. & McMaster, C. Developments in stubble retention in cropping systems in southern Australia. (*Department of Primary Industries*) <https://www.csu.edu.au/__data/assets/pdf_file/0009/922725/Developments-in-Stubble-Retention-in-Cropping-Systems-in-Southern-Australia-11Dec2013.pdf> (2013) (Date of access: 15/11/2015).

65 Bending, G. D. & Turner, M. K. *Incorporation of nitrogen from crop residues into light-fraction organic matter in soils with contrasting management histories* 45 thesis, (2009).

66 WA. Grazing stubbles and dry pasture. (*Department of Agriculture and Food, Government of Western Australia*) <https://www.agric.wa.gov.au/autumn/grazing-stubbles-and-dry-pasture> (2016) (Date of access: 19/72015).

67 Gustavsson, J., Cederberg, C., Sonesson, U., Van Otterdijk, R. & Meybeck, A. Global food losses and food waste. *Food and Agriculture Organization of the United Nations, Rom* (2011).

68 Brulliard, C. *et al.* The Australian recycling sector. (*Department of Sustainability, Environment, Water, Population and Communities*) <https://www.environment.gov.au/system/files/resources/dc87fd71-6bcb-4135-b916-71dd349fc0b8/files/australian-recycling-sector.pdf> (2012) (Date of access: 10/11/2015).

69 Smith, K., O’Farrell, K. & Brindley, F. Waste and recycling in Australia 2011: incorporating a revised method for compiling waste and recycling data. (*Australian Government Department of Sustainability, Environment, Water, Population and Communities*) <http://olr.npi.gov.au/wastepolicy/publications/pubs/waste-recycling2011.pdf> (2012) (Date of access: 6/5/2015).

70 Randell, P., Pickin, J. & Grant, B. Waste generation and resource recovery in Australia. (*Department of Sustainability, Environment, Water, Population and Communities*) <http://www.environment.gov.au/system/files/resources/4b666638-1103-490e-bdef-480581a38d93/files/wgrra.pdf> (2014) (Date of access: 26/12/2015).

71 Huynh, H. P. *Evaluating the use of alternative proteins in feed on growth and nutrient utilization of Australian catfish, Tandanus tandanus with emphasis on environmental contamination*, RMIT University, (2010).

72 Oita, A., Nagano, I. & Matsuda, H. An improved methodology for calculating the nitrogen footprint of seafood. *Ecological Indicators*. **60**, 1091-1103, doi:10.1016/j.ecolind.2015.08.039 (2016).

73 Farmery, A. K., Gardner, C., Green, B. S., Jennings, S. & Watson, R. A. Domestic or imported? An assessment of carbon footprints and sustainability of seafood consumed in Australia. *Environmental Science & Policy*. **54**, 35-43, doi:10.1016/j.envsci.2015.06.007 (2015).

74 Farmery, A., Gardner, C., Green, B. S., Jennings, S. & Watson, R. Life cycle assessment of wild capture prawns: expanding sustainability considerations in the Australian Northern Prawn Fishery. *Journal of Cleaner Production*. **87**, 96-104, doi:10.1016/j.jclepro.2014.10.063 (2015).

75 Stone, D. A., Allan, G. L., Parkinson, S. & Rowland, S. J. Replacement of fish meal in diets for Australian silver perch, Bidyanus bidyanus: III. Digestibility and growth using meat meal products. *Aquaculture*. **186**, 311-326 (2000).

76 Rowland, S. J. Review of aquaculture research and development of the Australian freshwater fish silver perch, Bidyanus bidyanus. *Journal of the World Aquaculture Society*. **40**, 291-324 (2009).

77 Booth, M. A., Allan, G. L., Frances, J. & Parkinson, S. Replacement of fish meal in diets for Australian silver perch, Bidyanus bidyanus: IV. Effects of dehulling and protein concentration on digestibility of grain legumes. *Aquaculture*. **196**, 67-85 (2001).

78 D. G. Bowley, G. L. A. Nutrients in Pond Based Aquaculture Discharge Water Used for Irrigation. (*NSW Department of Primary Industries, Port Stephens Fisheries Institute*) <http://www.dpi.nsw.gov.au/__data/assets/pdf_file/0003/638634/Bowley-and-Allan-Water-sampling-results-and-discuss-V2a.pdf> (2012) (Date of access: 06/09/2015)

79 Wiedemann, S., McGahan, E. J. & Poad, G. Using life cycle assessment to quantify the environmental impact of chicken meat production. Report No. 1742543847, (*Rural Industries Research and Development Corporation*) <https://rirdc.infoservices.com.au/items/12-029> (2012) (Date of access: 24/72015).

80 Bengtsson, J. & Seddon, J. Cradle to retailer or quick service restaurant gate life cycle assessment of chicken products in Australia. *Journal of Cleaner Production*. **41**, 291-300 (2013).

81 Eshel, G., Shepon, A., Makov, T. & Milo, R. Land, irrigation water, greenhouse gas, and reactive nitrogen burdens of meat, eggs, and dairy production in the United States. *Proceedings of the National Academy of Sciences*. **111**, 11996-12001 (2014).

82 Wiedemann, S. & McGahan, E. Environmental assessment of an egg production supply chain using life cycle assessment. (*Australian Egg Corporation Limited*) <https://www.aecl.org/dmsdocument/44> (2011) (Date of access: 6/10/2015).

83 Wiedemann, S., McGahan, EJ & Burger, M. Layer hen manure analysis report. (*Australian Egg Corporation Limited*) <https://www.aecl.org/assets/RD-files/Outputs-2/1EC082A-Final-Report.pdf> (2008) (Date of access: 17/7/2015).

84 Gourley, C., Aarons, S. & Hannah, M. Regularities in soil nutrient concentrations on dairy farms in Australia.

85 Unkovich, M. Nitrogen fixation in Australian dairy systems: review and prospect. *Crop and Pasture Science*. **63**, 787-804 (2013).

86 Gourley, C. J. P. *et al.* Farm-scale nitrogen, phosphorus, potassium and sulfur balances and use efficiencies on Australian dairy farms. *Animal Production Science*. **52**, 929, doi:10.1071/an11337 (2012).

87 Gourley, C. J., Aarons, S. R., Dougherty, W. J. & Weaver, D. M. in *Proceedings of 24th Annual Fertilizer and Lime Research Centre Workshop, Massey University.* 17.

88 Stephan, M. & Hobsbawn, P. Australian fisheries and aquaculture statistics 2014. (*Department of Agriculture and Water Resources*) <http://data.daff.gov.au/data/warehouse/9aam/afstad9aamd003/2014/AustFishAquacStats_2014_v1.0.0.pdf> (2014) (Date of access: 15/11/2015).

89 Farmery, A., Gardner, C., Green, B. S. & Jennings, S. Managing fisheries for environmental performance: the effects of marine resource decision-making on the footprint of seafood. *Journal of Cleaner Production*. **64**, 368-376 (2014).

90 Allan, G. L. *et al.* Replacement of fish meal in diets for Australian silver perch, Bidyanus bidyanus: I. Digestibility of alternative ingredients. *Aquaculture*. **186**, 293-310 (2000).

91 Allan, G. *et al.* Replacement of fish meal in diets for Australian silver perch, Bidyanus bidyanus: V. Least-cost formulation of practical diets. *Aquaculture*. **186**, 327-340 (2000).

92 Lim-Camacho, L. *et al.* Facing the wave of change: stakeholder perspectives on climate adaptation for Australian seafood supply chains. *Regional Environmental Change*. **15**, 595-606, doi:10.1007/s10113-014-0670-4 (2014).

93 Zabaniotou, A. & Kassidi, E. Life cycle assessment applied to egg packaging made from polystyrene and recycled paper. *Journal of Cleaner Production*. **11**, 549-559 (2003).

94 Cichota, R. & Snow, V. Ammonia Volatilisation from Grazed Pastures. (*Dairy Australia*) <http://fertsmart.dairyingfortomorrow.com.au/wp-content/uploads/2013/06/P5.00401-Volatilisation-Review-_Final-2012.pdf> (2012) (Date of access: 3/8/2015).

95 Stott, K. J. & Gourley, C. J. P. Intensification, nitrogen use and recovery in grazing-based dairy systems. *Agricultural Systems*. **144**, 101-112, doi:10.1016/j.agsy.2016.01.003 (2016).

96 Unkovich, M. Nitrogen fixation in Australian dairy systems: review and prospect. *Crop and Pasture Science*. **63**, 787, doi:10.1071/cp12180 (2012).

97 Birchall, S., Dillon, C. & Wrigley, R. Effluent and manure management database for the Australian dairy industry. (*Dairy Australia*) <http://www.dairyingfortomorrow.com.au/wp-content/uploads/combined.pdf> (2008) (Date of access: 11/2/2016).

98 Gourley, C., Powell, J., Dougherty, W. & Weaver, D. Nutrient budgeting as an approach to improving nutrient management on Australian dairy farms. *Animal Production Science*. **47**, 1064-1074 (2007).

99 ACMF. The Australian Chicken Meat Industry: An Industry in Profile. (*Australian Chicken Meat Federation (ACMF) Inc*) <http://www.chicken.org.au/industryprofile/downloads/The_Australian_Chicken_Meat_Industry_An_Industry_in_Profile.pdf> (2011) (Date of access: 10/9/2015).

100 Brunton, C. Chicken Meat Usage and Attitude Survey. (*Rural Industries Research and Development Corporation*) <http://www.chicken.org.au/files/_system/Document/Reports/RIRDC%20Report%20on%20Usage%20and%20Attitude%20Survey%202009.pdf> (2009) (Date of access: 10/6/2015).

101 TARGET100. Waste in Processing. (*Meat & Livestock Australia Limited*) <http://www.target100.com.au/Environment/Waste-Products/Waste-in-Processing> (2015) (Date of access: 11/12/2015).

102 Wiedemann, S., McGahan, E., Grist, S. & Grant, T. Environmental assessment of two pork supply chains using life cycle assessment. Report No. 174151973X, (*Rural Industries Research and Development Corporation*) <https://rirdc.infoservices.com.au/items/09-176> (2010) (Date of access: 30/6/2015).

103 Kruger, I., Mills, G. & Madden, P. PigGas–Pork industry greenhouse gas calculator and case studies. (*Australian Pork Limited and the Department of Agriculture*) <https://australianpork.infoservices.com.au/items/2009-1011335-REPORT> (2013) (Date of access: 15/1/2016).

104 Wolfe, E. Country pasture/forage resource profiles: Australia. (*Food and Agriculture Organization of the United Nations*) <http://www.fao.org/ag/agp/agpc/doc/counprof/PDF%20files/Australia.pdf> (2009) (Date of access: 15/1/2016).

105 Henry, B., Charmley, E., Eckard, R., Gaughan, J. B. & Hegarty, R. Livestock production in a changing climate: adaptation and mitigation research in Australia. *Crop and Pasture Science*. **63**, 191, doi:10.1071/cp11169 (2012).

106 Unkovich, M. J., Baldock, J. & Peoples, M. B. Prospects and problems of simple linear models for estimating symbiotic N2 fixation by crop and pasture legumes. *Plant and Soil*. **329**, 75-89, doi:10.1007/s11104-009-0136-5 (2009).

107 Geoff Duddy, C. S., Alan Bell, Roger Hegarty, Geoff Casburn. Feedlotting lambs. (*NSW Department of Primary Industries*) <http://www.dpi.nsw.gov.au/__data/assets/pdf_file/0020/193313/Feedlotting-lambs.pdf> (2016) (Date of access: 15/7/2015).

108 Wiedemann, S., Yan, M.-J. & Murphy, C. Resource use and environmental impacts from Australian export lamb production: a life cycle assessment. *Animal Production Science* (2015).

109 Henry, B. K., Butler, D. & Wiedemann, S. G. A life cycle assessment approach to quantifying greenhouse gas emissions from land-use change for beef production in eastern Australia. *The Rangeland Journal*. **37**, 273, doi:10.1071/rj14112 (2015).

110 Wiedemann, S., McGahan, E., Murphy, C. & Yan, M. Resource use and environmental impacts from beef production in eastern Australia investigated using life cycle assessment. *Animal Production Science*, doi:10.1071/an14687 (2015).

111 Wiedemann, S. G. *et al.* Resource use and greenhouse gas intensity of Australian beef production: 1981–2010. *Agricultural Systems*. **133**, 109-118, doi:10.1016/j.agsy.2014.11.002 (2015).

112 Wiedemann, S. & Yan, M. in *Proceedings of the 9th International Conference on Life Cycle Assessment in the Agri-Food Sector (LCA Food 2014), San Francisco, California, USA, 8-10 October, 2014.* 1512-1520 (American Center for Life Cycle Assessment).

113 Redding, M. *Quantifying Greenhouse Gas Emissions from Australian Piggeries*, Department of Employment, Economic Development and Innovation, (2008).

114 Skerman, A., Willis, S., McGahan, E., Borgognone, M. & Batstone, D. Validation of PigBal model predictions for pig manure production. *Animal Production Science*. **56**, 1081-1090 (2015).

115 Wiedemann, S. G. *et al.* Application of life cycle assessment to sheep production systems: investigating co-production of wool and meat using case studies from major global producers. *The International Journal of Life Cycle Assessment*. **20**, 463-476, doi:10.1007/s11367-015-0849-z (2015).

116 Henry, B. K., Butler, D. & Wiedemann, S. G. Quantifying carbon sequestration on sheep grazing land in Australia for life cycle assessment studies. *The Rangeland Journal*. **37**, 379, doi:10.1071/rj14109 (2015).

117 McFarland, I. Feeding and managing sheep in dry times. 2006) (Date of access: 11/2/2016).

118 Howden, S., White, D. & Bowman, P. Managing sheep grazing systems in southern Australia to minimise greenhouse gas emissions: adaptation of an existing simulation model. *Ecological modelling*. **86**, 201-206 (1996).

119 Bell, A. & Alcock, D. Full hand feeding of sheep–management. 4 2007) (Date of access: 11/4/2016).

120 Bell, L. W. & Moore, A. D. Integrated crop–livestock systems in Australian agriculture: Trends, drivers and implications. *Agricultural Systems*. **111**, 1-12 (2012).

121 MLA. A producers’ guide to production feeding for lamb growth. (Meat & Livestock Australia) 2007) (Date of access: 11/12/2015).

122 Wiedemann, S. *et al.* Environmental impacts and resource use of Australian beef and lamb exported to the USA determined using life cycle assessment. *Journal of Cleaner Production*. **94**, 67-75, doi:10.1016/j.jclepro.2015.01.073 (2015).

123 Desjardins, R. *et al.* Carbon Footprint of Beef Cattle. *Sustainability*. **4**, 3279-3301, doi:10.3390/su4123279 (2012).

124 McGinn, S. M. *et al.* Evaluating dispersion modeling options to estimate methane emissions from grazing beef cattle. *J Environ Qual*. **44**, 97-102, doi:10.2134/jeq2014.06.0275 (2015).

125 Capper, J. L. Is the Grass Always Greener? Comparing the Environmental Impact of Conventional, Natural and Grass-Fed Beef Production Systems. *Animals (Basel)*. **2**, 127-143, doi:10.3390/ani2020127 (2012).

126 Loh, Z. *et al.* Measurement of greenhouse gas emissions from Australian feedlot beef production using open-path spectroscopy and atmospheric dispersion modelling. *Australian Journal of Experimental Agriculture*. **48**, 244, doi:10.1071/ea07244 (2008).

127 Peters, G. M. *et al.* Red meat production in australia: life cycle assessment and comparison with overseas studies. *Environ Sci Technol*. **44**, 1327-1332, doi:10.1021/es901131e (2010).

128 Sun, J. *et al.* Effects of lignite application on ammonia and nitrous oxide emissions from cattle pens. *Sci Total Environ*. **565**, 148-154, doi:10.1016/j.scitotenv.2016.04.156 (2016).

129 Denmead, O. T. *et al.* Emissions of the indirect greenhouse gases NH3 and NOx from Australian beef cattle feedlots. *Australian Journal of Experimental Agriculture*. **48**, 213, doi:10.1071/ea07276 (2008).

130 Chen, D. *et al.* A new cost-effective method to mitigate ammonia loss from intensive cattle feedlots: application of lignite. *Sci Rep*. **5**, 16689, doi:10.1038/srep16689 (2015).

131 Bai, M. *et al.* Non-interference measurement of CH4, N2O and NH3 emissions from cattle. *Animal Production Science*, doi:<http://dx.doi.org/10.1071/AN14992> (2015).

132 Bai, M., Flesch, T. K., McGinn, S. M. & Chen, D. A Snapshot of Greenhouse Gas Emissions from a Cattle Feedlot. *J Environ Qual*. **44**, 1974-1978, doi:10.2134/jeq2015.06.0278 (2015).
